# A biomimetic 3D printed gradient tri-layered scaffold stimulating osteochondral differentiation of mesenchymal stem cells for mandibular condyle regeneration

**DOI:** 10.1186/s12903-026-09899-8

**Published:** 2026-10-09

**Authors:** Shaimaa H. Dawood, Dalia I. Sherief, Iman A. Fathy, Reem Magdy Khallaf, Dalia I. El-Korashy

**Affiliations:** 1 Department of Dental Biomaterials, Faculty of Dentistry, Ain Shams University, Organization of African Unity Street, Abbasia, Cairo, 11566 Egypt; 2 Department of Dental Biomaterials, School of Dentistry, Badya University, 6Th of October City, Giza, 3256201 Egypt; 3 Department of Oral Biology, Faculty of Dentistry, Ain Shams University, Organization of African Unity Street, Abbasia, Cairo, 11566 Egypt

**Keywords:** Tensile strength, Interfacial adhesion strength, Biodegradation, Cell differentiation, Immunofluorescence, Cell proliferation, Safranin O staining, Alizarin red staining

## Abstract

**Objectives:**

This study aimed to fabricate and assess the physical, mechanical, and in vitro biological properties of 3D printed single-layered scaffolds that simulate cartilage, calcified cartilage, and subchondral bone tissue. As well as a tri-layered osteochondral scaffold with a gradient composition and porosity.

**Methods:**

Six groups were fabricated using a masked stereolithography-based 3D printer. The cartilage group consisted of polylactic acid (PLA)/ 2% hyaluronic acid (HA) with the smallest pores and percentage porosity. The calcified cartilage group comprised PLA/ 2% HA/10% nano-hydroxyapatite (nHAp). Subchondral bone scaffold was formed of PLA/ 25% nHAp with the largest pore size and % porosity. The three scaffold designs were assembled to create the tri-layered group. There were two control groups. A total of 683 scaffolds were 3D printed. Tensile strength, compressive strength, moduli, and interfacial adhesion strength were evaluated at 24 h and 30 days. The biodegradation % of different scaffolds was assessed after 7, 14, and 28 days. Cell viability was assessed through cell counting, cell adhesion, and H & E staining. Cell differentiation was assessed using fast green/safranin O, alizarin red staining, and immunofluorescence after coculturing with rat bone marrow mesenchymal stem at 7, 14, and 21 days. Data were statistically analyzed at *p* ≤ 0.05.

**Results:**

The tensile strengths and moduli of the PLA/2%HA and PLA/25% nHAp scaffolds resembled those of normal cartilage and trabecular bone, respectively. The tri-layered scaffold exhibited strong interfacial adhesion strength and demonstrated 13.68% weight loss after 30 days. The tri-layered scaffold exhibited a stiffness gradient, supporting biological responses consistent with chondrogenic differentiation on the more flexible side of the scaffold and osteogenic differentiation on the stiffer side. It demonstrated evidence of both chondrogenic and osteogenic differentiation.

**Conclusions:**

Incorporation of HA and nHAp, as well as variations in porosity, significantly impacted scaffolds' properties.

**Clinical relevance:**

These findings suggested the potential of the fabricated scaffolds for medical and maxillofacial regenerative applications, particularly for repairing cartilage, bone, and osteochondral defects.

**Supplementary Information:**

The online version contains supplementary material available at https://doi.org/10.1186/s12903-026-09899-8.

## Introduction

The mandibular condyle of the temporomandibular joint (TMJ) plays an important role in daily functions, such as chewing and speaking. It facilitates articulation with the TMJ disc and reduces stress concentration on the underlying bone. Damage to the joint can seriously impact patients’ quality of life, and its management imposes a significant global economic burden each year [1, 2]. The mandibular condyle comprises osteochondral (OC) tissue that includes three distinct regions with continuous gradient structure: cartilage, calcified cartilage, and subchondral bone.

Mandibular condylar cartilage is classified as secondary cartilage and differs fundamentally from the primary articular cartilages found in other synovial joints, like the knee. Unlike articular cartilage, which develops embryonically as hyaline cartilage and is composed predominantly of type II collagen and proteoglycans, the condylar cartilage develops postnatally in response to biomechanical stimuli. It consists of a superficial part of fibrocartilage (fibrous zone) and a deeper part of hyaline cartilage, which is formed from proliferative, mature, and hypertrophic zones. The fibrous zone consists of fibroblasts and organized collagen I. The proliferative zone contains mesenchymal stem cells that differentiate into fibroblasts and chondrocytes. The mature or chondroblastic zone is made of collagen II and various proteoglycans. The deepest layer, the hypertrophic zone, expresses collagen X [3, 4]. Aggrecan is the predominant type of proteoglycan present in mandibular condylar cartilage extracellular matrix [5, 6].

Treatment of osteochondral defects necessitates simultaneous regeneration of mandibular condylar cartilage and subchondral bone tissue. The primary challenge lies in the differences between cartilage and bone in terms of extracellular matrix (ECM) and mechanical properties [1]. Bone is a highly regenerative tissue owing to the presence of blood supply, nerves, and lymphatics. Hydroxyapatite, the main component of natural bone, is the most applied calcium phosphate in osteochondral regeneration. It demonstrates excellent osteoconductivity, osteoinductivity, and extreme biocompatibility [7–9]. In contrast, mandibular condylar cartilage lacks blood supply, nerves, and lymphatics [2, 3, 10]. Hyaluronic acid (HA) is a key component of cartilage ECM. It plays a critical role in regulating cell functions. It facilitates cell migration, proliferation, and differentiation of mesenchymal stem cells into chondrocytes. It increases the level of chondrogenic protein expression, which is required for cartilage regeneration. It can link aggrecan molecules to form larger proteoglycans. HA is an attractive biomaterial for fabricating cartilaginous scaffolds due to its bioactive properties, biomimetic structure, good biocompatibility, appropriate degradation rate, and high printability [11–13]. Polylactic acid (PLA), a synthetic polymer, is an aliphatic thermoplastic polyester, commonly used in 3D printed scaffolds and approved by the FDA in 1970 for use in biological tissues [14, 15]. It is characterized by its adequate mechanical properties, ease of production, and high reproducibility in additive manufacturing techniques. The chain ends of PLA can be easily functionalized, facilitating their integration with other chemical groups, allowing for simpler use in UV-curable systems, such as vat polymerization-based 3D printing techniques [15]. However, PLA possesses some drawbacks, such as its slow degradation rate, hydrophobicity, and low cell affinity. Sometimes an inflammatory response can occur from the acidity of degradation products. These drawbacks can be minimized by blending PLA with natural polymers or bioceramics [14, 16].

Osteochondral scaffold design has evolved from mono-layered to bi-layered and multi-layered designs, attempting to mimic natural tissue. Mono-layered scaffolds lack structural and compositional heterogeneity. Bi-layered scaffolds feature cartilage and bone regeneration. However, tri-layered scaffolds involved the intermediate calcified cartilage layer [17, 18].

Scaffold-based methods have emerged as promising strategies, aiming to replicate the native tissue [19]. Conventional methods such as salt leaching, gas foaming, solvent casting, phase separation, and freeze-drying are used in the fabrication of scaffolds for tissue engineering. However, these methods make it very difficult to control pore geometry, size, and distribution. Besides, they are characterized by poor repeatability [18]. Additive manufacturing techniques have become popular in tissue engineering. It enables the creation of three-dimensional scaffolds with specific architectures and controlled porosities [20–22].

In the 2020 s, liquid crystal display (LCD)-based 3D printing technology, a variant of masked stereolithography (MSLA) vat polymerization, has dominated the market due to its ability to combine high resolution, faster production, and lower cost compared to digital light processing technologies [17, 18, 23, 24].

Various obstacles still hinder osteochondral regeneration using 3D printed multi-layered scaffolds. Mimicking the biophysical and biomechanical gradients of native osteochondral tissue proves challenging. Although some scaffolds include gradients, their capacity to replicate the zonal heterogeneity of cartilage and subchondral bone remains limited. Often, scaffolds do not meet the mechanical demands of load-bearing roles while also maintaining biocompatibility and bioactivity [25, 26]. A key challenge is establishing a strong, stable bond between scaffold layers, as delamination or mechanical failure at interfaces is common, especially under joint loading [26]. Moreover, few studies have examined the interfacial strength of these multi-layered scaffolds [26, 27]. There is a need for advanced 3D printing techniques capable of producing complex scaffolds with high precision and reproducibility. Ongoing research should focus on developing materials, designs, and fabrication methods that balance mechanical strength with biological functionality, with the goal of creating functionally integrated osteochondral scaffolds.

Accordingly, this study aimed to fabricate, characterize, and evaluate 3D-printed single-layered scaffolds that simulate cartilage, calcified cartilage, and subchondral bone tissues of the mandibular condyle, as well as their combination in a tri-layered osteochondral scaffold with gradient composition and porosity, to mimic the native heterogeneity of osteochondral tissue using an MSLA-LCD-based vat polymerization 3D printer with innovative tilt-release technology. This technology involves tilting the resin tank downward by one layer thickness after printing each layer, enabling rapid peeling of the scaffold from the vat film after curing, thereby reducing separation between scaffold layers during 3D printing and producing an integrated structure.

The first null hypothesis tested was that there would be no difference in the interfacial adhesion strength of the tri-layered scaffold after 24 h and 30 days. The second null hypothesis was that the different compositions and porosities of the scaffolds would not affect the compressive strength, tensile strength, elastic modulus, biodegradation %, in vitro cell proliferation, chondrogenic differentiation, and osteogenic differentiation over different time intervals.

## Materials and methods

A photosensitive polylactic acid (PLA) (eResin PLA Pro, eSUN Industrial Co., Ltd, Shenzhen, China, 840,249,100,839), containing trimethylbenzoyldiphenyl phosphine oxide photoinitiator, urethane, and epoxy acrylates essential for photocuring, was used. Nano-hydroxyapatite (nHAp) (Nano gate, Cairo, Egypt, 202,103) of 100 nm ± 20 nm length, and 10–20 nm width was used. Hyaluronic acid (HA) (Acros Organics Co., Ltd, New Jersey, USA, A0405554) was supplied as 95% sodium hyaluronate.

### Determination of the optimized concentration of nHAp within the PLA, preparation of different scaffold solutions

PLA, HA, and nHAp were mixed at room temperature using a magnetic stirrer in different concentrations. In the current study, the concentration of nHAp was optimized in the PLA photopolymer for PLA/nHAp subchondral bone scaffold, as the bioactivity of bone scaffolds increased by increasing the concentration of hydroxyapatite [28]. The nano-hydroxyapatite powder was mixed with PLA at concentrations of 40 wt%, 30 wt%, and 25 wt% to obtain a homogeneous dispersion. The optimal concentration was determined according to the results of the 3D printing trials. The 3D printing solutions with concentrations of more than 25% were highly viscous, with the precipitation of some of the nHAp. These precipitates interfered with the 3D printing process, leading to impossible printing and perforation of the vat sheet.

According to the manufacturer, each 100 g of eResin PLA pro liquid contains 20 g of pure PLA. These 20 g were considered the total amount (100%) of the PLA that was used in proportioning of different solutions. Mass fraction and mixing time of PLA, nHAp, and HA for each 100 g eResin PLA pro liquid are listed in Table 1.

**Table 1:** Mass fraction (wt% (g)) and mixing time of PLA, nHAp, and HA for each 100 g eResin PLA pro liquid

**Solution type**	**PLA wt% (g)**	**nHAp wt% (g)**	**HA wt% (g)**	**Final formulation (wt %/wt %)**	**Stirring time**
Control groups solution	100% (20 g)	Zero%	Zero%	100% PLA	-
Cartilage (C) solution	98% (19.6 g)	Zero %	2% (0.4 g)	98% PLA/2% HA	One hour
Calcified cartilage (CC) solution	88% (17.6 g)	10% (2 g)	2% (0.4 g)	88% PLA/10% nHAp/2% HA	Two hours
Subchondral bone (SB) layer solution	75% (15 g)	25% (5 g)	Zero %	75% PLA/25% nHAp	Four hours

The stirring time was determined based on a pilot study upon obtaining a homogenous mixture with no precipitates

### Scaffold design and 3D printing process

Three different CAD models were designed using SolidWorks 2020 software according to the size and percentage porosity with a hexagonal pore geometry [15, 29]. After designing each of the single-layered scaffolds separately, they were assembled to produce a tri-layered interconnected design of gradient porosity. The three layers of the tri-layered scaffold were designed with equal thickness to ensure methodological consistency and maintain balanced structural representation of the cartilage, calcified cartilage, and subchondral bone regions. The composition, grouping, pore size and % porosity of the fabricated scaffolds are shown in Table 2. The CAD files of different scaffolds were saved as Standard Triangle Language (STL) files. Then, CHITUBOX V.1.9.5 slicer software was used to manipulate the STL files, add the bottom support structure of the scaffolds, and to adjust the printing parameters, as shown in Table 3. The processed file was saved as G-code and loaded into the 3D printer via a flash memory.

**Table 2:** Composition, grouping, pore size and % porosity of different scaffolds

**Group number**	**Group name**	**Description**	**Composition (wt%)**	**(Pore size (µm)& percentage porosity (%)**
1	PLA 200	Cartilage (C) control single-layered scaffold	100% PLA	200 µm & 50%
2	PLA 400	Subchondral bone (SB) control single-layered scaffold	100% PLA	400 µm & 70%
3	PLA/HA	Cartilage (C) single-layered scaffold	98% PLA/2% HA	200 µm & 50%
4	PLA/HA/nHAp	Calcified cartilage (CC) single-layered scaffold	88% PLA/2% HA/10% nHAp	300 µm & 60%
5	PLA/nHAp	SB single-layered scaffold	75% PLA/25% nHAp	400 µm & 70%
6	Tri-layered C/CC/SB scaffold	Tri-layered scaffold	Top C layer 98% PLA/2% HA ** + ** Intermediate CC layer 88%PLA/2%HA/10%nHAp ** + ** Bottom SB layer 75%PLA/25% nHAp	Top C layer (200 µm & 50%) ** + ** Intermediate CC **(**300 µm & 60%) ** + ** Bottom SB layer (400 µm & 70%)

**Table 3:** Printing parameters for the fabrication of scaffolds of different groups

Layer thickness	0.030 mm
Bottom layer (base structure) count	6 layers
Bottom exposure time	35 s
Exposure time of the different groups	1.5 to 3.5 s
Transition layer count	0
Transition type	Linear
Light off delay	6 s
Bottom light off delay	2 s

An MSLA-LCD-based 3D printer (ELEGOO Saturn 4 Ultra, 12 K, China) was used. It reveals an innovative tilt-release technology that involves tilting the resin tank downward by one layer thickness distance after printing each layer, and the platform is lifted upwards, enabling rapid peeling of the scaffold from the vat film after curing, thus reducing the separation between the layers of the scaffold during the 3D printing process.

For the tri-layered scaffold, upon completing the 3D printing of the cartilage part, the printing was paused, the vat was emptied, and refilled with the CC solution. The vat was returned to its position, the exposure time was adjusted for the new material, and the printing was resumed. The new part was photocured at the bottom of the cartilage part. The process was paused again, and the same steps were repeated for printing the SB part on the bottom of the CC. After 3D printing, the scaffolds were washed by immersion in 99% ethanol, sonicated (3 min) to remove uncured resin, and post-cured for 6 min.

### Sample size calculation

Sample size was calculated using G* power 3.1.9.6 software with a power value of 80%, and α set at 0.05, based on an internal pilot study. A total of 683 samples were performed. In this study, the primary outcomes are interfacial adhesion strength and immunofluorescence tests. For interfacial adhesion strength, an effect size of 1.96 at 80% statistical power indicated a required sample size of 6 specimens per group. To further increase the statistical power, the sample size was increased to 7 specimens, and this sample size was also used for tensile strength, compressive strength and biodegradation % tests. For immunofluorescence, an effect size of 5.604 at 80% statistical power indicated a required sample size of 3 specimens per group, which was further increased to 5 specimens per group to provide additional statistical power. This sample size was used for cell proliferation and histochemical staining as well. Regarding the variance assumption, homogeneity of variance was assumed and assessed in SPSS. It was found that Sig. (*p*) > 0.05, indicating that the assumption of homogeneity of variance was achieved.

### Structural characterization and chemical analysis of the fabricated scaffolds

Scaffold shrinkage was assessed by measuring the diameter and height using a digital caliper to calculate the % change in volume relative to the CAD model. Samples were gold-sputtered and examined by SEM (JEOL, JCM-6000 Plus, USA) to evaluate pore size, geometry, and connectivity. Energy dispersive X-ray (EDX) in conjunction with SEM was used for elemental analysis. A Fourier transform infrared spectrometer (FT-IR) with an attenuated total reflectance (ATR) (Burker Alpha II, Ettlingen, Germany) was used to analyze the functional groups of the fabricated scaffolds.

### Determination of the percentage of porosity of the 3D printed scaffolds

The real percentage of porosity of the scaffolds after 3D printing was determined by a modified liquid displacement method. PLA 200, PLA 400, PLA/HA cartilage, PLA/HA/nHAp CC, and PLA/nHAp SB groups were evaluated. A total of 15 cylindrical specimens of 10 mm diameter and 3 mm thickness were tested (*n* = 3). Each scaffold was immersed in 5 mL of ethanol for 10 min until the air overflowed completely. The volume of ethanol was measured before and after immersion and was recorded as V1 and V2, respectively. The scaffold was removed, and the residual ethanol volume was recorded as V3. The percentage porosity (% P) was calculated as follows [30, 31]:

$$\%\;\mathrm{P}=\frac{v1-v3}{v2-v3} \times 100\%$$

### Compressive strength and modulus of elasticity

**Fig. 1:**
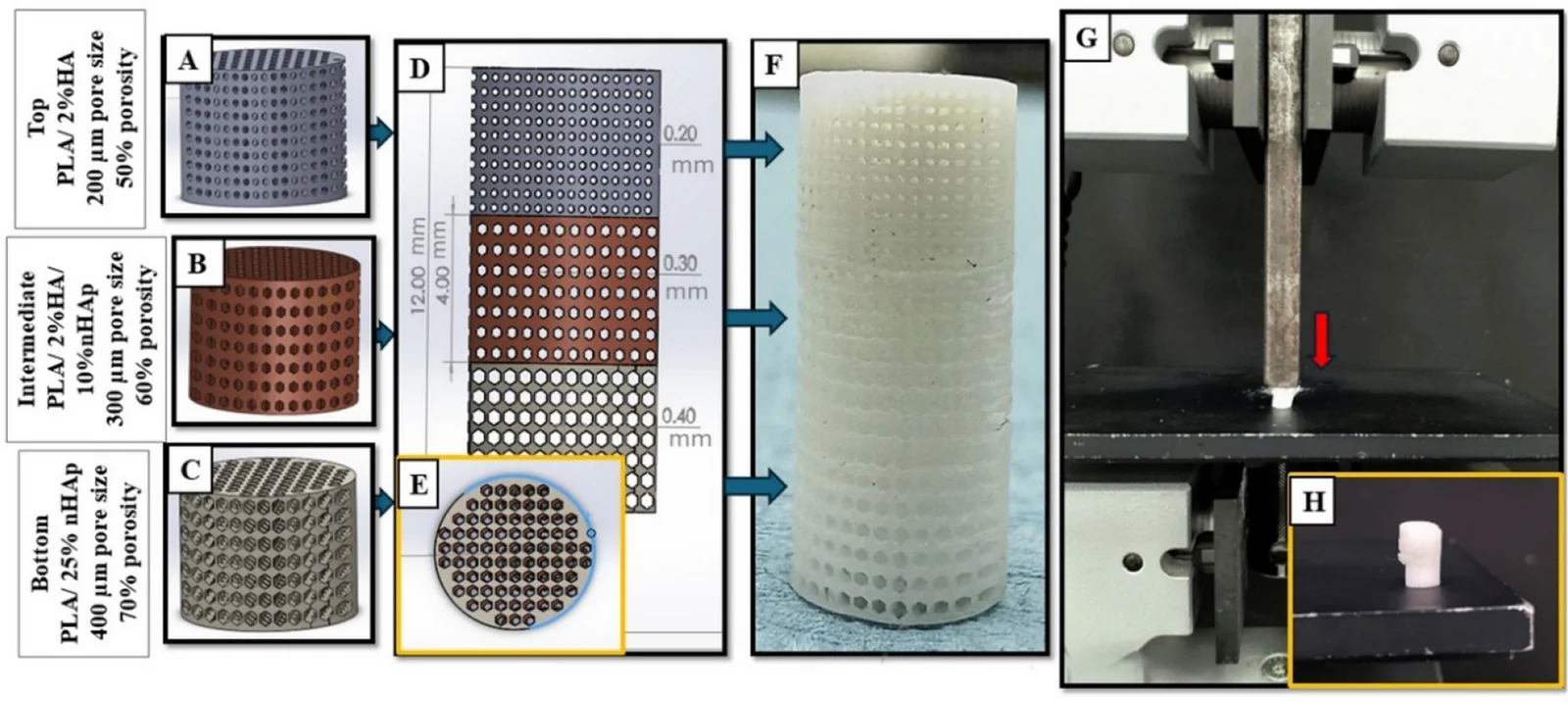
**A**, **B**, **C** 3D design of the top, intermediate, and bottom layers of the tri-layered scaffold for compressive strength testing, respectively. **D** The assembly of the tri- C/CC/SB scaffold with gradient porosities, **E**. The top-view image, revealing the interconnectivity of the pores along the scaffold structure, **F**. An image of the integrated 3D printed tri-layered scaffold, **G**. Compressive strength testing image, showing the direction of load, **H**. The tri-layered scaffold after maximum load, revealing the ability of the scaffold to recover and hence its high flexibility

All scaffold groups were prepared and tested according to ISO 844:2021 [32]. Cylindrical scaffolds of 12 mm length and 6 mm diameter were 3D printed. Each group was divided into two subgroups (*n* = 7) according to testing time at 24 h and 30 days. Samples were stored in phosphate-buffered saline (PBS) at pH 7.4 and 37 °C. The PBS was replaced weekly. Each scaffold was subjected to loading to fracture using a universal testing machine (UTM) (Instron TM 3365, Massachusetts, UK) with a 5 kN load cell at a crosshead speed of 1 mm/min. The loading direction was perpendicular to the printing direction of the scaffold to avoid failure, which may be induced by weak interlayer bonding (Fig. 1) [33].

### Tensile strength & elastic modulus

**Fig. 2:**
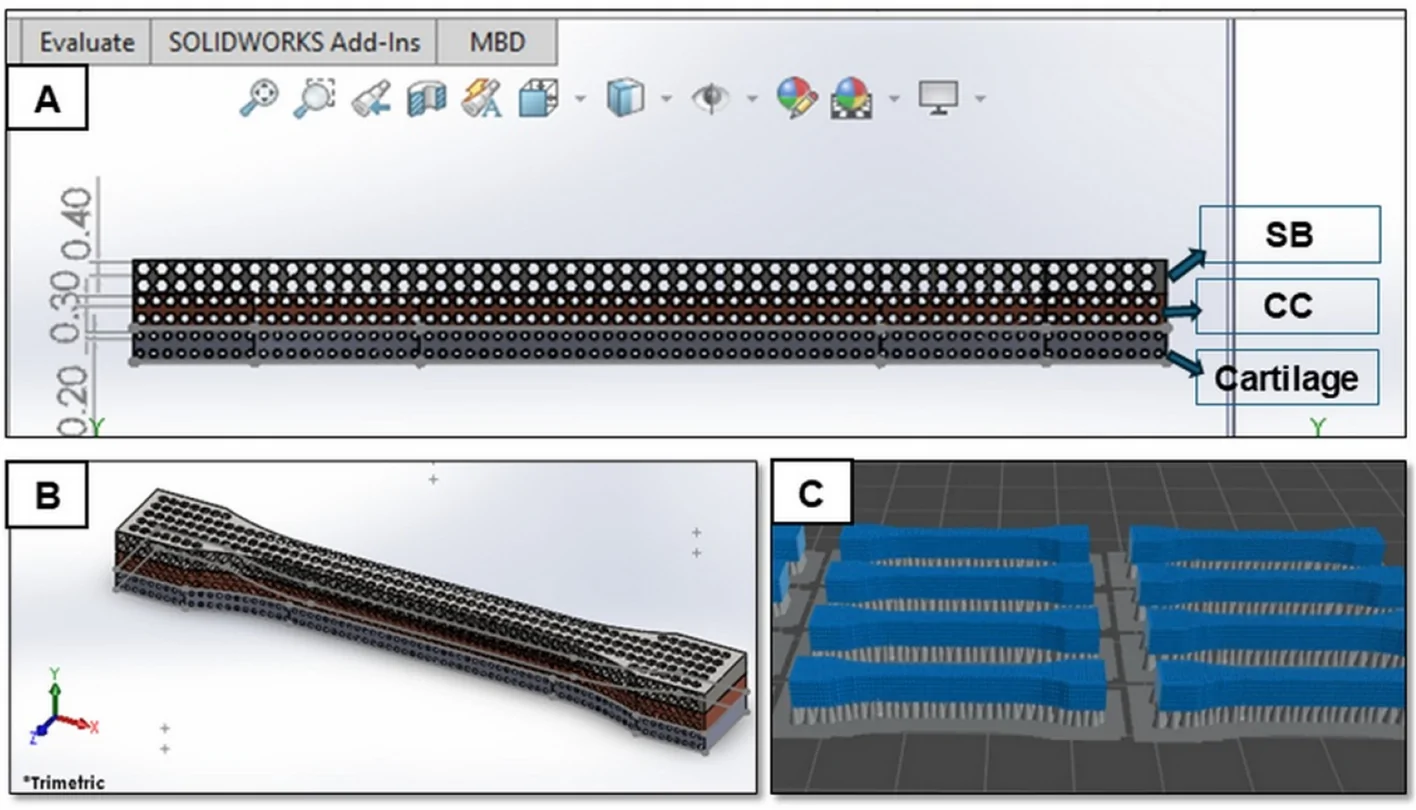
Images showing tri-layered scaffolds for tensile strength and modulus testing, **A**, **B**. the design of the scaffold: a side view and 3D view, respectively, **C**. An image showing the direction of printing

Scaffolds were prepared and tested according to ISO 527-2-2018 [34]. A total of 84 dumbbell-shaped scaffolds from all groups were 3D printed. The tri-layered C/CC/SB specimen design was divided horizontally into three equal parts of cartilage, calcified cartilage, and subchondral bone, each 1 mm in thickness, as shown in Fig. 2.

Each group was divided into two subgroups (*n* = 7) based on the storage time (24 h, 30 days). Samples were stored in phosphate-buffered saline (PBS) at 37 °C with pH 7.4. The PBS was replaced weekly. Before testing, each specimen was marked at both ends to monitor any slippage between the fixture and the sample. Each scaffold was subjected to tensile loading up to fracture using UTM at a crosshead speed of 1 mm/min. The loading direction was parallel to the layer-printing direction [33].

### Interfacial adhesion strength

**Fig. 3:**
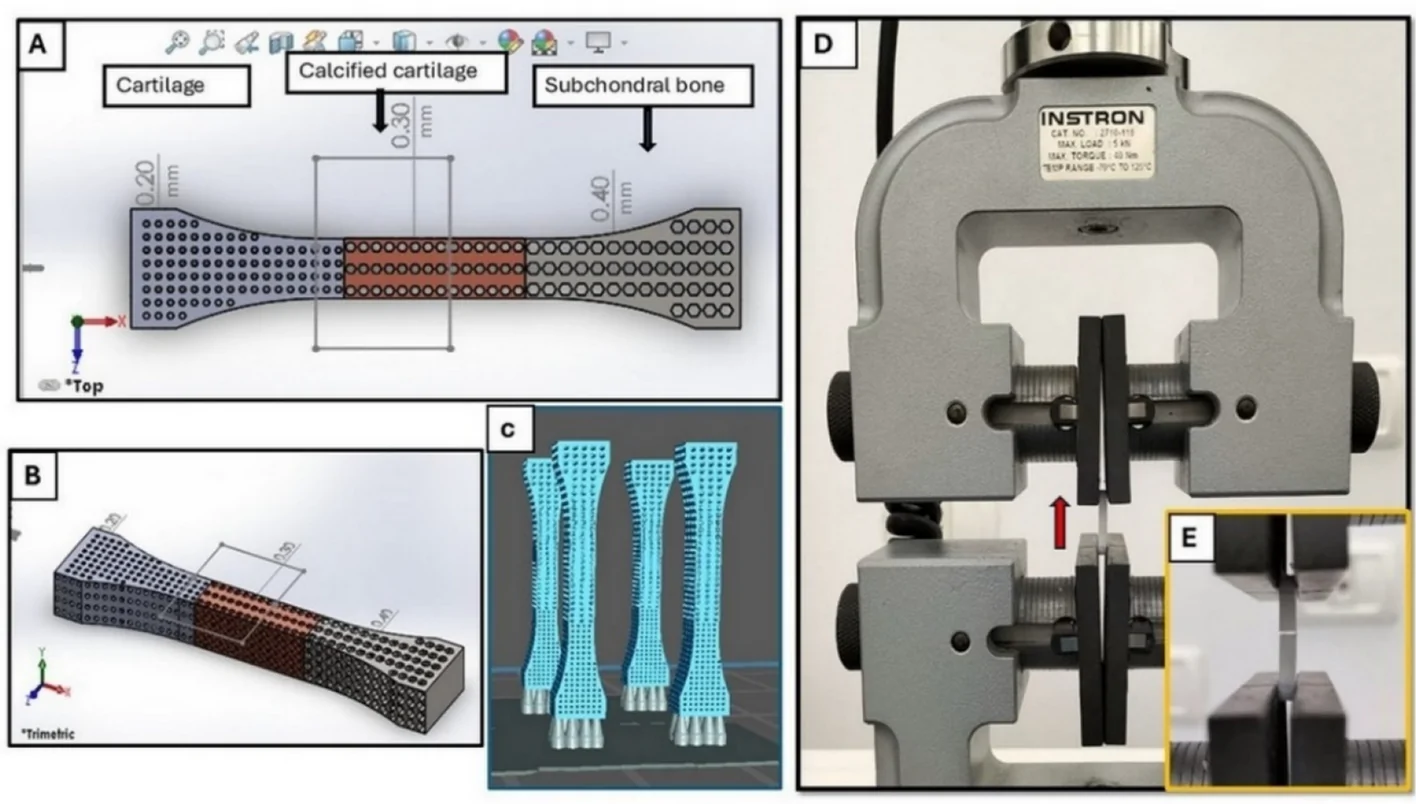
**A**, **B** Images, showing the top view (**A**) and 3D view (**B**) of the assembly structure of the tri-layered scaffold design with gradient porosity for interfacial adhesion strength testing, **C** An image showing the direction of printing. **D** The interfacial adhesion strength testing, showing the direction of loading, **E** The fractured sample image

Scaffolds were prepared and tested according to ISO 527-2-2018 [34]. A total of 14 tri-layered C/CC/SB scaffolds were designed in a dumbbell shape. Each specimen was designed with its length divided into three equal parts, representing the lower subchondral bone, the intermediate calcified cartilage, and the upper cartilage layers (Fig. 3). They were stored and subdivided as in the tensile strength testing [35]. Each scaffold was subjected to tensile loading up to failure using the UTM at 1 mm/min crosshead speed with the load direction applied perpendicular to the printing direction to facilitate separation between the layers. Fractured scaffolds were gold-sputtered and observed by SEM to determine the failure mode.

### In vitro evaluation of biodegradation (weight loss%)

The scaffolds were prepared and tested according to ISO 13781–2017 [36]. Each group was divided into three subgroups based on testing time at 7, 14, and 30 days (*n* = 7). The scaffolds were sterilized before testing by immersion in 70% ethanol (30 min) and then left under UV for 2 h to avoid bacterial contamination [36]. The initial weight (W0) was recorded. Each specimen was then soaked in 10 mL of PBS (pH 7.4) in a sealed tube at 37°C. The PBS was replaced weekly. At each interval, the specimens were removed from the PBS, transferred to a desiccator containing silica gel, and connected to a vacuum pump at 100 mbar (Vacuubrand, ME 1, Germany) until a dry constant mass was reached (W1) [11].

$$\mathrm{Weight}\;\mathrm{loss}\;\%=\left({\mathrm W}_0-{\mathrm W}_1\right)/{\mathrm W}_0\times100.$$

#### Biological evaluation

Rat bone marrow mesenchymal stem cells (rBMMSCs) of newborn female albino Sprague Dawley rats (50 g) were purchased from Central Lab of Stem Cells and Biomaterials Applied Research (CLSBAR), Faculty of Dentistry, Ain Shams University. Before experimental use, rBMMSCs were phenotypically characterized. Flow cytometric characterization demonstrated that the isolated cells exhibited a mesenchymal stem cell immunophenotype. The cells showed high expression of the positive MSC markers CD90 (97.34%) and CD73 (94.22%), while displaying very low expression of the hematopoietic marker CD45 (1.02%). The autofluorescence control showed negligible background signals (FITC: 0.08%, PE: 0.41%). These findings confirm that the analyzed cell population possesses the characteristic surface marker profile of mesenchymal stem cells, being positive for CD90 and CD73 and negative for CD45 (Additional file 1). Cells were suspended in complete culture medium (CCM) in T-75 flasks and incubated at 37 °C and 5% CO_2_. Each 100 mL of CCM was prepared from 80 mL of high-glucose Dulbecco's Modified Eagle Medium (DMEM) with stable L-glutamine and sodium pyruvate, 15 mL of fetal bovine serum, and 5 mL of antibiotic/antimycotic (Anti-Anti). After reaching confluence and at passage 2, cells were seeded on the scaffolds.

#### Cell viability

The assessment of cell viability of the fabricated scaffolds included evaluation of cell proliferation using trypan blue staining and cell counting at 1, 3, and 7 days. Cell adhesion capability of all groups was observed after 7 days of coculturing with rBMMSCs using SEM. Finally, cell viability was assessed by hematoxylin and eosin staining to evaluate cellular infiltration and attachment at 7,14, and 21 days.

Cylindrical discs (4.5 mm diameter, 3 mm thickness) of PLA 200, PLA 400, PLA/HA cartilage, PLA/HA/nHAp CC, PLA/nHAp SB, and tri-layered C/CC/SB groups were prepared. The pore size and percentage porosity of each group were maintained as specified in Table 2. Each group was divided into three subgroups based on culturing time at 1, 3, and 7 days. The scaffolds were sterilized by soaking in 70% ethanol for 30 min and left under constant UV-light exposure for 2 h. The scaffolds were then immersed in phosphate buffer saline for 2 h to remove residual ethanol [37]. After sterilization, the scaffolds were placed in a 96-well plate and incubated in CCM at a cell density of 1 × 10^5^ per scaffold.

For cell proliferation assessment, cells were recovered from the porous scaffolds before trypan blue counting. After retrieving the scaffolds from the culture medium, they were washed with PBS to remove residual medium and non-adherent cells. Adherent cells were then detached by incubation with 0.25% trypsin–EDTA at 37 °C for 2 min, with gentle pipetting to facilitate cell release from the porous architecture. The resulting cell suspension was collected, and the scaffolds were subsequently rinsed with fresh culture medium to recover any remaining detached cells. The collected fractions were pooled and centrifuged at 600 rpm for 4 min. The cell pellet was suspended in culture medium and mixed with trypan blue solution for cell proliferation assessment. Viable cells were counted using an automated cell counter (Countess II, Thermo Fisher Scientific, MA, USA) [37, 38].

#### Assessment of in-vitro osteogenic and chondrogenic differentiation

Cylindrical scaffolds (14 mm diameter, 3 mm thickness) were 3D printed [38]. Each group was divided into 3 subgroups based on the culturing time at 7, 14, and 21 days. They were placed into 6-well plates at a cell density of 1 × 10^5^ per scaffold and cultured in a medium of DMEM with stable L-glutamine and sodium pyruvate with 10% fetal bovine serum, 40 μg/ml dexamethasone, 50 μg/ml L-ascorbic acid, and 120 µg/ml antibiotic/antimycotic, which were renewed every 2 days [39]. For the tri-layered C/CC/SB group, 1 × 10^5^ cell suspension was seeded with a chondral layer upward for 3 h, then the scaffold was inverted, and another 1 × 10^5^ cell suspension was seeded [30].

Fast green/safranin O (S.O) histochemical staining (Alfa Aesar, Massachusetts, USA, 477–73-6) was used for PLA 200, PLA/HA cartilage, PLA/HA/nHAp CC, and tri-layered C/CC/SB group (*n* = 5). Fast green/S.O positive staining demonstrated the formation of glycosaminoglycan matrix of the cartilage, which stained orange to red, while the nuclei were stained black [30, 38, 40]. Immunofluorescence (IF) testing with the aggrecan rabbit pAb (Abclonal, Massachusetts, USA, 5500019919), which corresponds to amino acids 420–575 of human aggrecan, was used to confirm chondrogenic differentiation in the positively stained groups (*n* = 5). Aggrecan is one of the proteoglycans in mandibular condylar cartilage [30, 38].

Alizarin red histochemical staining (Loba Chemie PVT. LTD, Tarapur, India, SL31052010) was used for PLA 400, PLA/nHAp SB, PLA/HA/nHAp CC, and tri-layered C/CC/SB groups (*n* = 5) to evaluate the ECM mineralization [30, 40–42]. The calcium deposits were stained orange to red. Acellular scaffold specimens were subjected to identical Alizarin Red staining procedures and used as controls to assess potential interactions between the dye and scaffold-associated calcium-containing components (Additional file 2). The IF of osteocalcin (BGLAP) Rabbit mAb (Abclonol, Massachusetts, USA, 3523060114), which has a sequence corresponding to amino acids 1–95 of human osteocalcin, was done in positively stained groups (*n* = 5). Osteocalcin is a protein expressed in mature osteoblasts during the late phases of osteogenic differentiation and is a calcium-binding protein that is part of the organic bone matrix [30, 40, 42].

#### Preparation of the cultured scaffolds for staining and the immunofluorescence test

Scaffolds were removed from the medium on 7, 14, and 21 days, fixed with 10% paraformaldehyde (40 min), and washed three times with PBS for 3 min each [40]. The single- layered scaffolds of PLA 200, PLA 400, PLA/HA, PLA/HA/nHAp, and PLA/nHAp were subjected directly to staining without sectioning. In contrast, the tri-layered scaffolds were frozen in liquid nitrogen for 15 min, then longitudinally cut with a surgical blade to expose the three layers. Conventional paraffin embedding of the tri-layered scaffolds was initially attempted; however, significant scaffold loss and structural distortion occurred during tissue processing, preventing reliable preservation of the scaffold architecture. The PLA polymer has been reported to be susceptible to faster degradation when exposed to higher temperatures approaching its Tg, and the degradation occurs by chain scission rather than hydrolysis [43, 44]. Therefore, liquid-nitrogen-assisted longitudinal sectioning was employed to maintain scaffold integrity and allow assessment of staining distribution throughout the constructs.

For immunofluorescence testing, each scaffold was treated with a permeabilization buffer containing 1% Triton X-100 (20 min) at room temperature, then rinsed three times with PBS for 3 min each. Then, it was treated with a blocking buffer containing 1% bovine serum albumin at room temperature for one hour and then aspirated. The primary antibodies, osteocalcin or aggrecan, diluted 1:50 (primary antibody: PBS), were added and incubated at 4 ^o^ C overnight in a dark condition. The corresponding fluorochrome-labeled secondary antibody, FITC-conjugated Goat anti-Rabbit IgG (H + L) (Abclonol, Massachusetts, USA), diluted 1:200 (Secondary antibody: PBS), was dropped for one hour in a moist environment in the dark [40]. Finally, the scaffold was observed by a confocal laser microscope (CLSM). The fluorochrome-labeled secondary antibody was excited at 491 nm, and the emission was 516 nm.

The IF intensity was quantified by gray value analysis using ImageJ software. To minimize the influence of scaffold autofluorescence on fluorescence quantification, cell-free scaffolds were included as background controls for each scaffold type (Additional files 3–4). All control and experimental samples were imaged using identical confocal microscope settings, including laser power, detector gain, pinhole size, and exposure parameters. The mean fluorescence intensity measured from the empty scaffolds was considered the baseline scaffold signal and was subtracted from the corresponding fluorescence values obtained from cell-seeded scaffolds. The resulting corrected values were used for all quantitative analyses and statistical comparisons.

For fluorescence quantification, a region of interest (ROI) encompassing the scaffold cross-sectional area was selected from each image using ImageJ software. The same ROI selection criteria were applied to all images within each experimental group. Images were converted to grayscale, and mean gray values were measured using ImageJ. To ensure consistency, identical image acquisition and analysis parameters were maintained throughout the study. Background-corrected fluorescence intensity values were subsequently used for statistical analysis.

### Statistical analysis

Data were tested for normality using Kolmogorov-Smirnov and Shapiro-Wilk tests. Two-way and one-way ANOVA tests were used to compare between groups, followed by Tukey’s post hoc test for pairwise multiple comparisons. Independent t-test and Chi-square test were used to compare the groups in the interfacial adhesion strength test. The significance level was set at *p* ≤*0.05*. Statistical analysis was performed with SPSS 23.0 (Chicago, IL, USA).

## Results

### Structural and chemical characterization

**Fig. 4:**
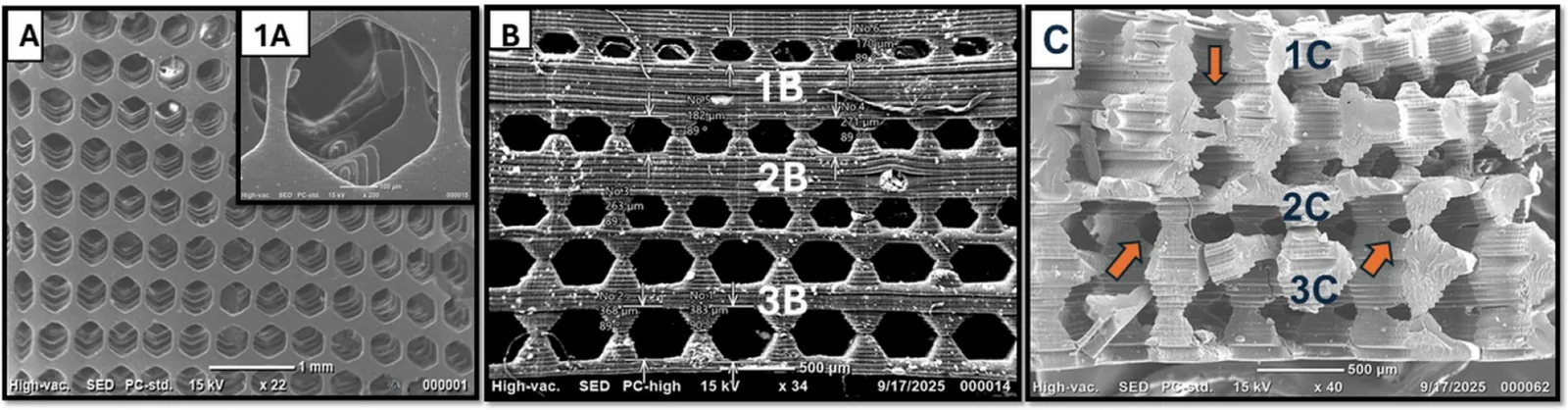
**A** 1 A SEM photomicrographs of the PLA 400 control group, showing highly porous and interconnected structure, **B** SEM photomicrographs of the tri-layered scaffold (side view), revealing the three different layers of the scaffold with gradient porosity, 1B. The top PLA/HA cartilage layer, 2B. The intermediate PLA/HA/nHAp calcified cartilage layer, 3B. The bottom PLA/nHAp subchondral bone layer, **C** Longitudinal section of tri-layered scaffold showing the three parts of the scaffold, respectively demonstrating the highly porous scaffold with interconnected porosities (red arrows) (N.B: the horizontal lines in (**B**, **C**) represent the 3D printing layers-deposition)

**Fig. 5:**
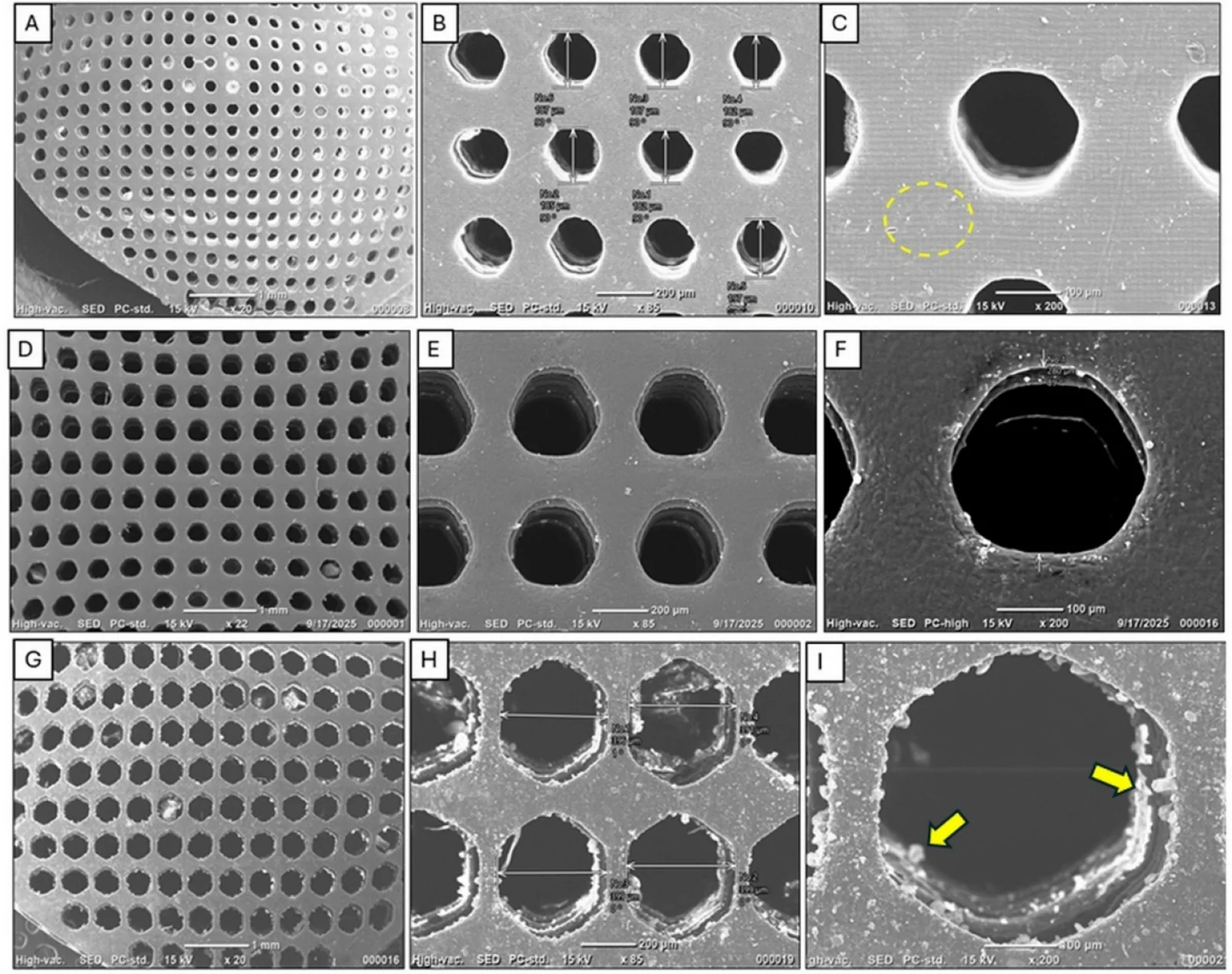
SEM photomicrographs of the single-layered scaffolds: **A** The PLA/HA cartilage scaffold, **B**, **C** Higher magnifications, showing homogenous dispersion of the HA particles (dotted circle), **D** The PLA/HA/nHAp CC scaffold, E, F. Higher magnifications, showing nHAp particles, that are more intense around the pore margins, **G** The PLA/nHAp SB scaffold, **H**, **I** Higher magnifications, showing more exposed nHAp particles and pronounced irregularities (yellow arrows)

**Fig. 6:**
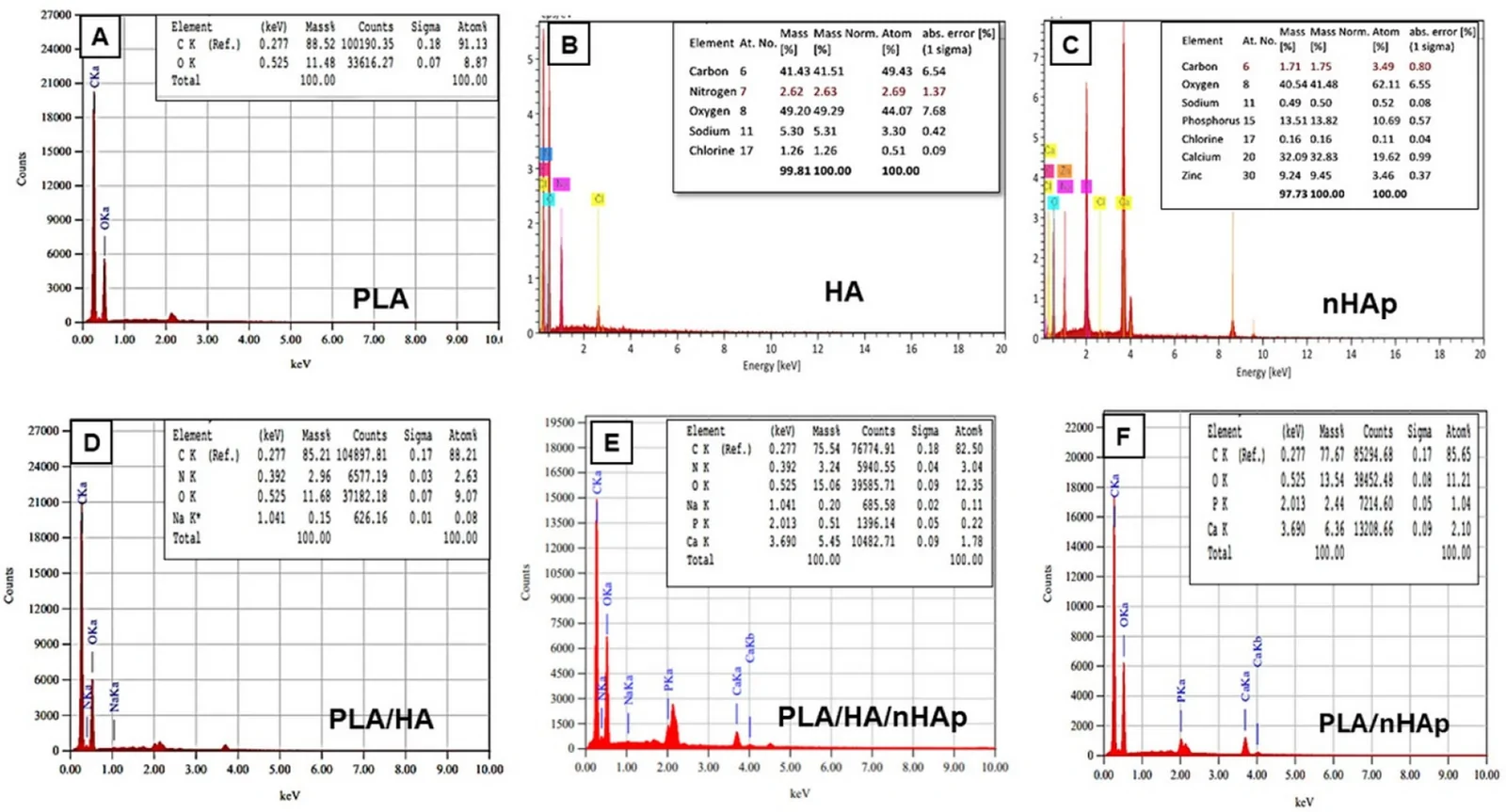
EDX spectra of the different groups, showing distribution of elements (Atomic%): **A** PLA showing the principle peaks of C and O, **B** HA showing the characteristic peaks of C, O, N, and Na, **C** nHA showing the main peaks of Ca, P, and O, **D** PLA/HAp scaffold showing the peaks of C, N, O and Na, **E** PLA/HA/nHAp scaffold showing the main peaks of C, N, O, Na, P, and Ca, **F** PLA/nHAp scaffold showing characteristic peaks of C, O, P, and Ca

After 3D printing, all scaffolds exhibited a volume shrinkage of 1.51 ± 0.38% compared to the original CAD model design. Scanning electron micrographs revealed the hexagonal pore geometry and confirmed that all groups had a highly porous and interconnected structure, as shown in Figs. 4 and 5. The high concentration of nHAp particles in the PLA/nHAp SB scaffold led to the appearance of more irregularities over the pore margins, increasing surface area/volume ratio, as shown in Fig. 5 G, H, I. The Scanning electron micrographs of the tri-layered C/CC/SB group revealed the three different layers of the scaffold with gradient composition and porosity (Figs. 4B, 3C). The comparison between the designed pore size and the actual measurement on SEM images revealed that all scaffolds experienced a slight reduction in pore size compared to their CAD models, with large reduction in the PLA/HA/nHAp and the PLA/nHAp groups, as shown in Table 4. The distribution of elements in the different materials and scaffolds (Atomic%), as determined by EDX analysis, is shown in Fig. 6.

**Table 4:** The pore size and % porosity of the different scaffolds on the CAD model and after 3D printing

**Group**	**Pore size on CAD model**	**Pore size after 3D printing**	**% porosity on CAD model**	**% porosity after 3D printing**
PLA 200	200 µm	197. 74 ± 3.6 µm	50%	49.2 ± 0.53%
PLA 400	400 µm	396.2 ± 3.8 µm	70%	68.63 ± 0.55%
PLA/HA cartilage	200 µm	180.2 ± 9.7 µm	50%	47.27 ± 1.10%
PLA/HA/nHAp CC	300 µm	265 ± 11.52 µm	60%	56.77 ± 1.25%
PLA/nHAp SB	400 µm	360 ± 23.33 µm	70%	64.43 ± 0.47%

**Fig. 7:**
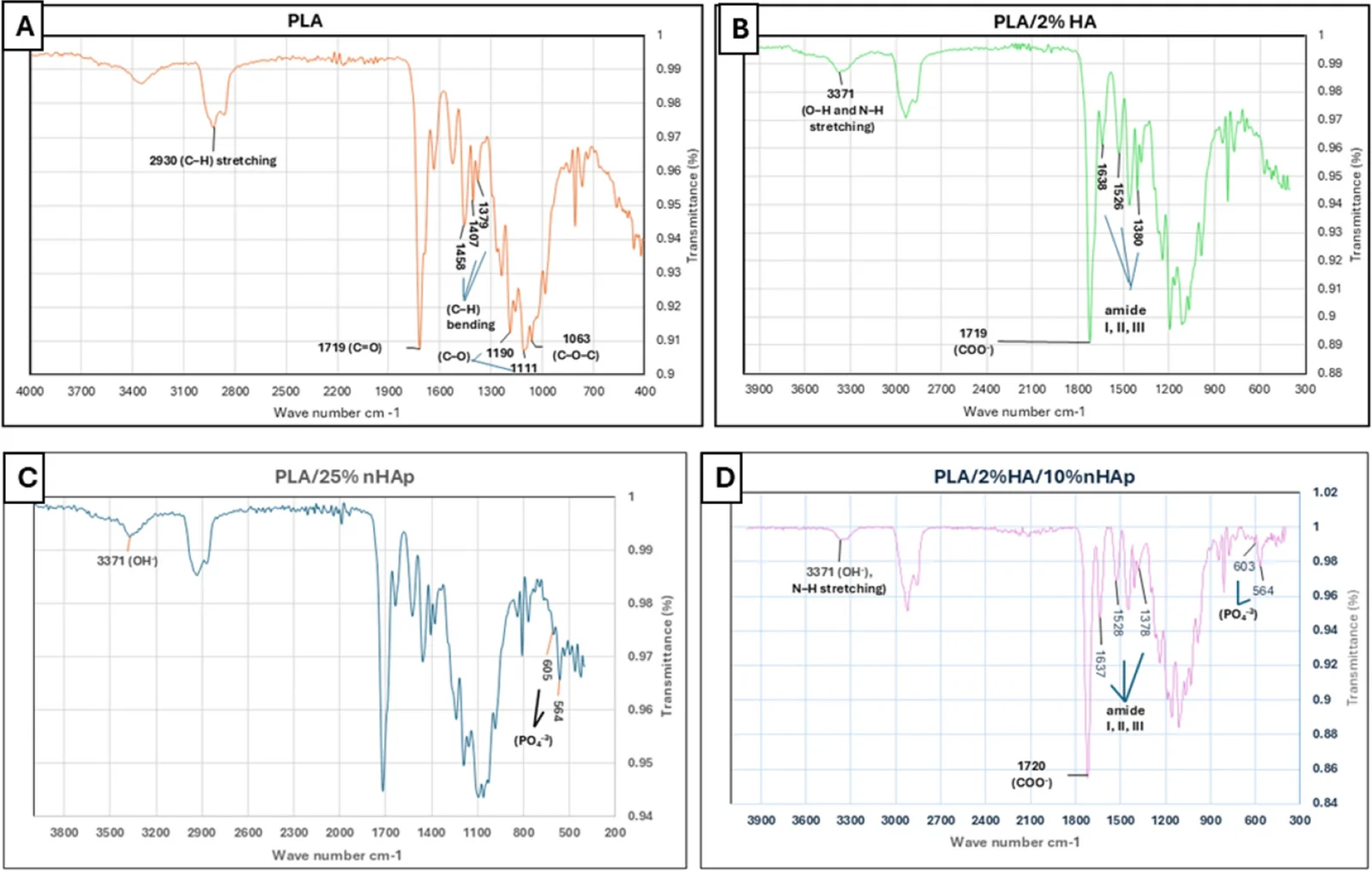
The ATR-FTIR spectra of the different scaffolds: **A** The PLA group, **B** The PLA/HA cartilage group, **C** The PLA/HA/nHAp CC group, **D** The PLA/nHAp SB group

FTIR spectrum of the PLA groups exhibited characteristic C–H stretching at 2930 cm⁻^1^, C–H bending at 1458, 1407, and 1379 cm⁻^1^, and C–O stretching near 1190 and 1111 cm⁻^1^. The peak at 1063 cm⁻^1^ indicated C–O–C stretching, while the band near 1719 cm⁻^1^ corresponded to C = O stretching. The PLA/nHAp SB group showed the same peaks as the PLA group with distinct OH absorption at 3371 cm⁻^1^, along with phosphate peaks at 564 and 605 cm⁻^1^ [45]. The PLA/HA cartilage group showed PLA peaks, as well. In addition, it showed hydrogen-bonded O–H and N–H near 3371 cm⁻^1^, and amide I, II, and III bands at 1638, 1526, and 1380 cm⁻^1^, respectively, with a carboxylate peak near 1719 cm⁻^1^ [46]. The PLA/HA/nHAp CC group combined the peaks present in the cartilage group and those present in the SB group (Fig. 7).

### Determination of the percentage porosity of the fabricated scaffolds

The comparison between the designed percentage porosity in the CAD model and its actual measurement after 3D printing revealed that all scaffolds exhibited a reduction in percentage porosity relative to their CAD models (Table 4).

### Compressive strength & modulus

**Fig. 8:**
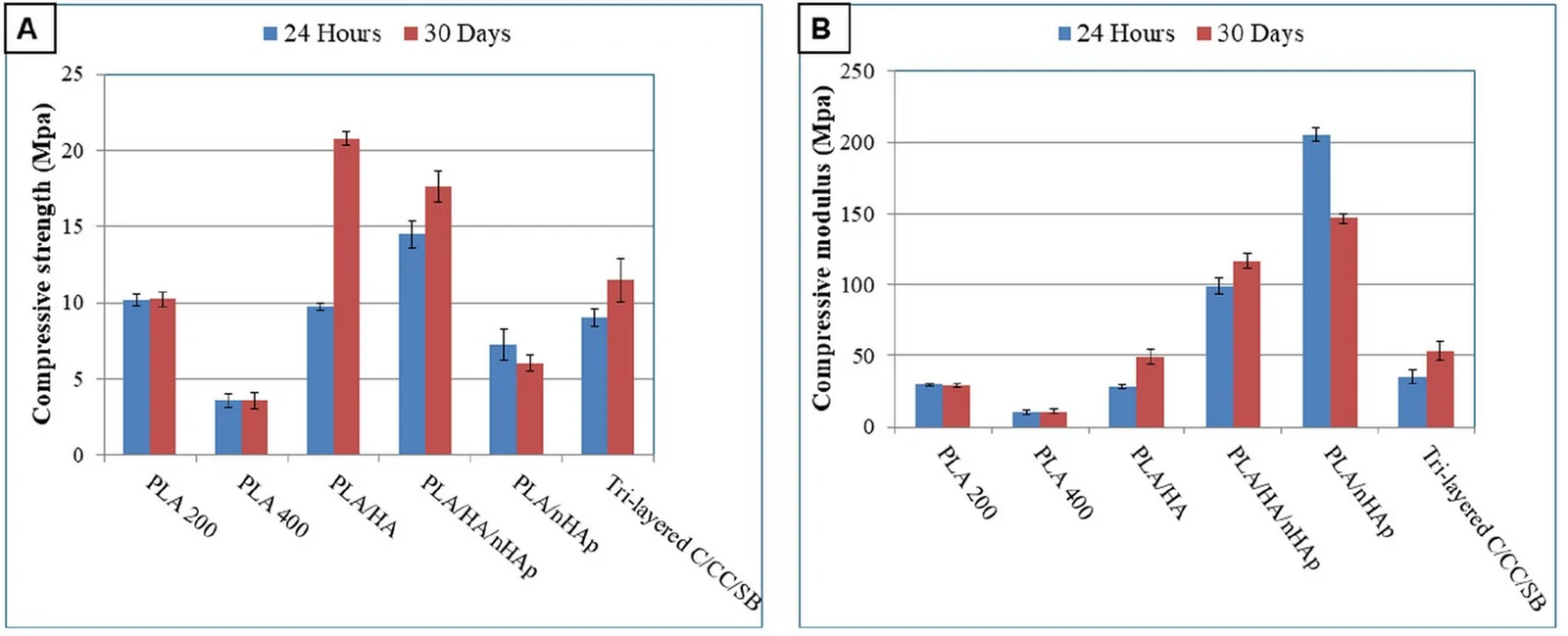
**A** Bar chart showing the mean compressive strength (MPa) and SD (Error bars) values of the groups at different aging intervals, **B** Bar chart showing the mean compressive modulus (MPa) and SD (Error bars) values of the groups at different aging intervals

Two-way ANOVA followed by Turkey’s post hoc test showed statistically significant differences in compressive strength and modulus among the scaffold groups and between aging periods at *p* < 0.005, as shown in Table 5 and Fig. 8. There was significant interaction between groups and aging time, at *p* = 0.001.

**Table 5:** Mean ± SD values, and significance of compressive strength (MPa), and compressive modulus (MPa) of different groups at 24 h and 30 days

**Groups**	**Compressive strength**		***p*-value**	**Compressive modulus**		***p*-value**
	**24 Hours**	**30 Days**		**24 Hours**	**30 Days**	
PLA 200	10.20 ± 0.35^B^	10.23 ± 0.49^C^	0.900	29.45 ± 1.09^D^	29.09 ± 1.11^D^	0.253
PLA 400	3.59 ± 0.46^E^	3.55 ± 0.52^E^	0.868	10.52 ± 1.74^F^	10.89 ± 1.64^E^	0.678
PLA/HA	9.74 ± 0.22^C^	20.79 ± 0.46^A^	0.001*	27.85 ± 1.58^E^	49.04 ± 5.30^C^	0.001*
PLA/HA/nHAp	14.46 ± 0.89^A^	17.58 ± 1.03^B^	0.002*	98.80 ± 5.30^B^	116.65 ± 5.40^B^	0.001*
PLA/nHAp	7.24 ± 1.04^D^	6.03 ± 0.53^D^	0.044*	205.24 ± 4.76^A^	146.55 ± 3.44^A^	0.001*
Tri- layered C/CC/SB	9.02 ± 0.57^C^	11.50 ± 1.41^C^	0.001*	35.19 ± 4.71^C^	53.19 ± 6.79^C^	0.001*
*p*-value	0.001*	0.001*		0.001*	0.001*	

Different uppercase superscript letters indicate a significant difference between groups in each age at (*p* < 0.05)

Significance level at *p* ≤ 0.05, *significant difference

PLA/HA cartilage group exhibited the highest increase in the compressive strength value at 30 days compared to 24 h. Also, the PLA/HA/nHAp CC, and the tri-layered C/CC/SB groups showed an increase in compressive strength after 30 days. In contrast, the PLA/nHAp SB group demonstrated a statistically significant decrease in compressive strength after 30 days compared to 24 h. While both PLA 200 and PLA 400 groups showed no significant difference between the two-time intervals. Regarding the compressive modulus, the PLA/nHAp SB group showed a significant reduction in compressive modulus over time. On the other hand, the PLA/HA/nHAp CC group, Tri-layered C/CC/SB, and PLA/HA cartilage groups showed significantly increased values at 30 days compared to 24 h. In contrast, both the PLA 200 and PLA 400 groups exhibited no significant differences between the two-time intervals.

### Tensile strength & modulus

**Fig. 9:**
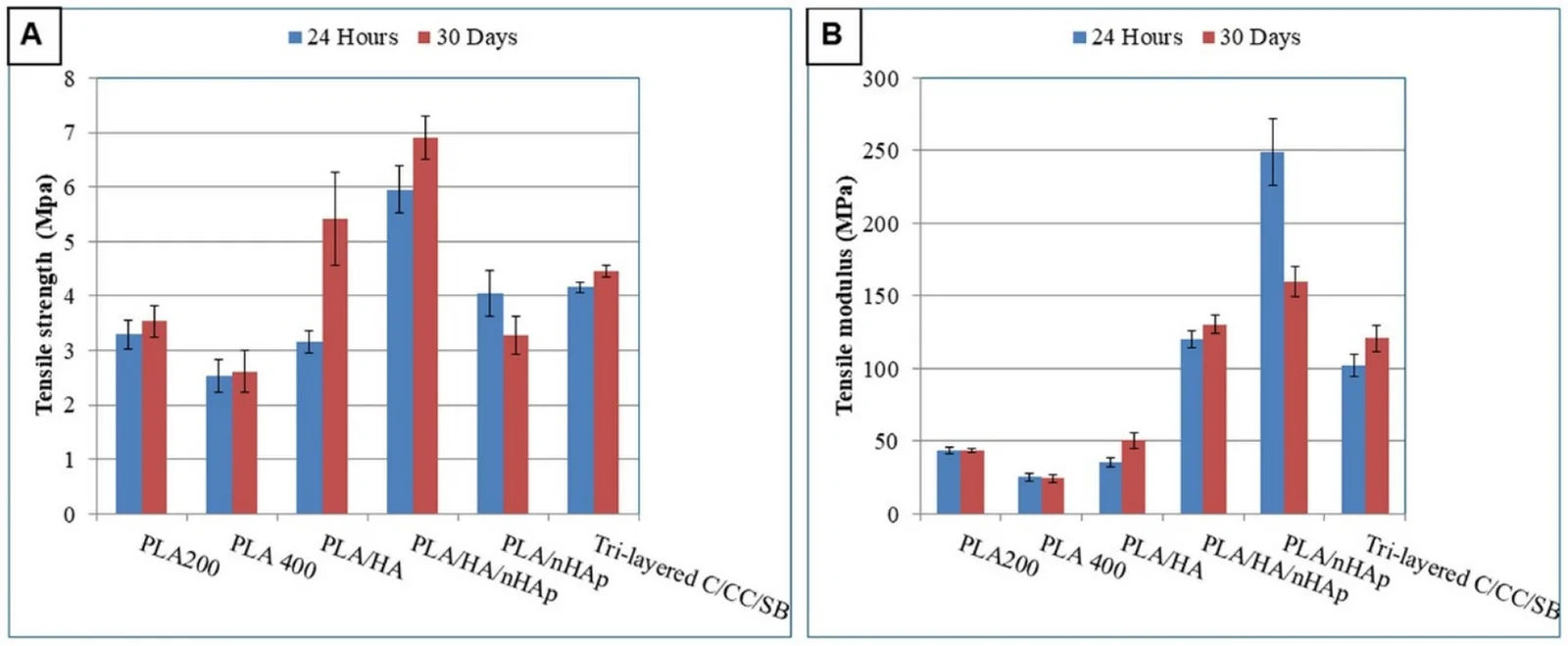
**A** Bar chart showing the mean Tensile strength (MPa) and SD (Error bars) values of the groups at different aging intervals, **B** Bar chart showing the mean Tensile modulus (MPa) and SD (Error bars) values of the groups at different aging intervals

Two-way ANOVA followed by Tukey’s post hoc test showed statistically significant differences in tensile strength and modulus among the different scaffold groups and between aging periods at *p* < 0.005, as shown in Table 6, Fig. 9. There was significant interaction between groups and aging time, at *p* = 0.001. The effect of aging on the tensile strength and modulus of different scaffolds showed the same pattern as in compressive strength and modulus results.

**Table 6:** Mean ± SD values, and significance of tensile strength (MPa), and tensile modulus (MPa) of different groups at 24 h and 30 days

**Groups**	**Tensile strength**		***p*-value**	**Tensile modulus**		***p*-value**
	**24 Hours**	**30 Days**		**24 Hours**	**30 Days**	
PLA 200	3.29 ± 0.25^C^	3.53 ± 0.28^D^	0.146	43.57 ± 2.34^D^	43.82 ± 1.18^E^	0.752
PLA 400	2.54 ± 0.30^D^	2.61 ± 0.38^E^	0.668	25.12 ± 2.90^F^	24.57 ± 2.67^F^	0.659
PLA/HA	3.16 ± 0.21^C^	5.41 ± 0.85^B^	0.001*	35.60 ± 3.07^E^	50.39 ± 5.23^D^	0.001*
PLA/HA/nHAp	5.95 ± 0.43^A^	6.90 ± 0.39^A^	0.015*	120.36 ± 5.87^B^	130.44 ± 6.28^B^	0.027*
PLA/nHAp	4.05 ± 0.42^B^	3.27 ± 0.35^D^	0.022*	248.86 ± 23.05^A^	160.16 ± 10.45^A^	0.001*
Tri-layered C/CC/SB	4.16 ± 0.09^B^	4.45 ± 0.11^C^	0.001*	102.29 ± 7.59^C^	120.92 ± 8.90^C^	0.018*
p-value	0.001*	0.001*		0.001*	0.001*	

Different uppercase superscript letters indicate a significant difference between groups in each age at (*p* < 0.05)

Significance level *p* ≤ 0.05, *significant

### Interfacial adhesion strength

**Fig. 10:**
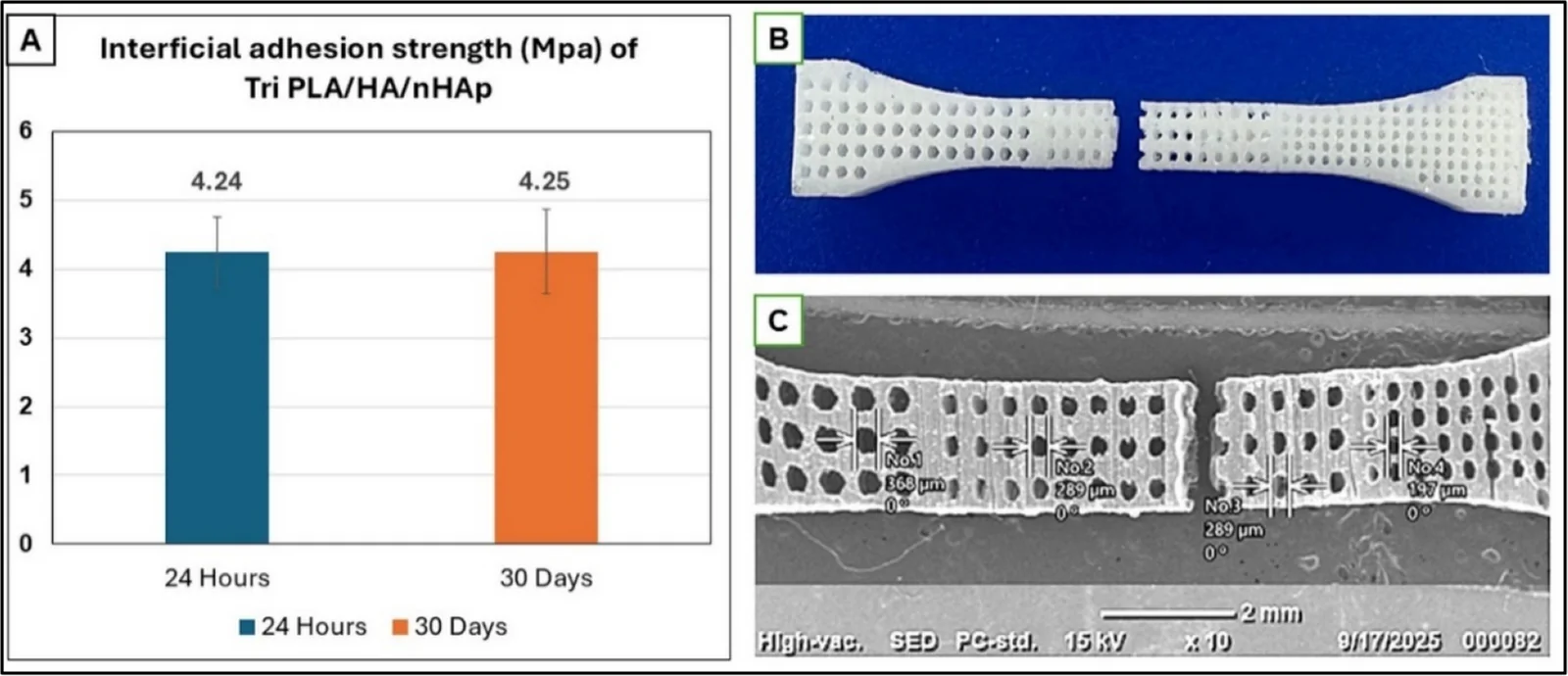
**A** Bar chart showing the mean interfacial adhesion strength (MPa) and SD (Error bars) values of the Tri-layered C/CC/SB group at 24 h and 30 days, **B** An image showing a fractured sample after interfacial adhesion strength testing, **C** Scanning electron micrograph of the tri-layered fractured sample revealing cohesive failure at the intermediate calcified cartilage (PLA/HA/nHAp) layer

The independent t-test showed no significant difference in the interfacial adhesion strength of the tri-layered C/CC/SB group at 24 h (4.24 ± 0.51) and 30 days (4.25 ± 0.61) at *p* = 0.980 (Fig. 10A).

The scanning electron micrographs revealed that the mode of failure was cohesive, and no adhesive failures were observed (Fig. 10B, C). The chi-square test revealed no significant difference between the distribution of failure modes after 24 h and 30 days’ time intervals, as shown in Table 7.

**Table 7:** Chi-square test showing the number of samples (No.) and percentage of modes of failure at 24 h and 30 days

**Mode of failure**	**Aging**				***p*-value**
	**24 Hours**		**30 Days**		
	**No**	**%**	**No**	**%**	
Cohesive in the cartilage layer	0	0.0%	0	0.0%	
Cohesive in the calcified cartilage layer	6	85.7%	7	100.0%	0.317
Cohesive in the subchondral bone layer	1	14.3%	0	0.0%	
Adhesive failure	0	0.0%	0	0.0%	
Total	7	100.0%	7	100.0%	

Significant at *p* ≤ 0.05, *significant

### In vitro evaluation of biodegradation (weight loss%)

Two-way ANOVA showed significant differences in the biodegradation (%) among the different groups at *p* = 0.001 and between different storage times at *p*** = **0.001. There was significant interaction between groups and storage time, at *p* = 0.001, as shown in Table 8. All scaffold groups showed an increase in weight loss by aging, with significant differences between the three storage times of 7, 14, and 30 days, except for PLA 200 and PLA 400, which exhibited no weight loss over the 30-day storage period. The tri-layered C/CC/SB group showed the most pronounced weight loss % from 5.986 ± 0.227 at 7 days to 13.681 ± 0.391% at 30 days.

**Table 8:** Mean ± SD values, and significance of weight loss (%) of different groups at 7, 14, and 30 days

**Groups**	**Weight loss%**			***p*-value**
	**7 days**	**14 days**	**30 days**	
PLA 200	0.00 ± 0.00^** Da**^	0.00 ± 0.00^**Ea**^	0.00 ± 0.00^**Ea**^	1.000
PLA 400	0.00 ± 0.00^** Da**^	0.00 ± 0.00^**Ea**^	0.00 ± 0.00^**Ea**^	1.000
PLA/HA	0.34 ± 0.02^**Cc**^	3.33 ± 0.09^**Bb**^	3.85 ± 0.05^**Ba**^	0.001*
PLA/HA/nHAp	1.97 ± 0.09^**Bc**^	2.45 ± 0.05^**Cb**^	2.76 ± 0.04^**Ca**^	0.001*
PLA/nHAp	0.00 ± 0.00^**Dc**^	0.79 ± 0.07^**Db**^	1.68 ± 0.12^** Da**^	0.001*
Tri-layered C/CC/SB	5.98 ± 0.22^**Ac**^	10.12 ± 0.610^**Ab**^	13.68 ± 0.39^**Aa**^	0.001*
*p*-value	0.001*	0.001*	0.001*	

Different uppercase superscript letters indicate a significant difference between groups in each age at (*p* < 0.05)

Different lowercase superscript letters indicate a significant difference between aging in each group (*p* < 0.05)

Significance level *p* ≤ 0.05, *significant

### Cell proliferation

**Fig. 11:**
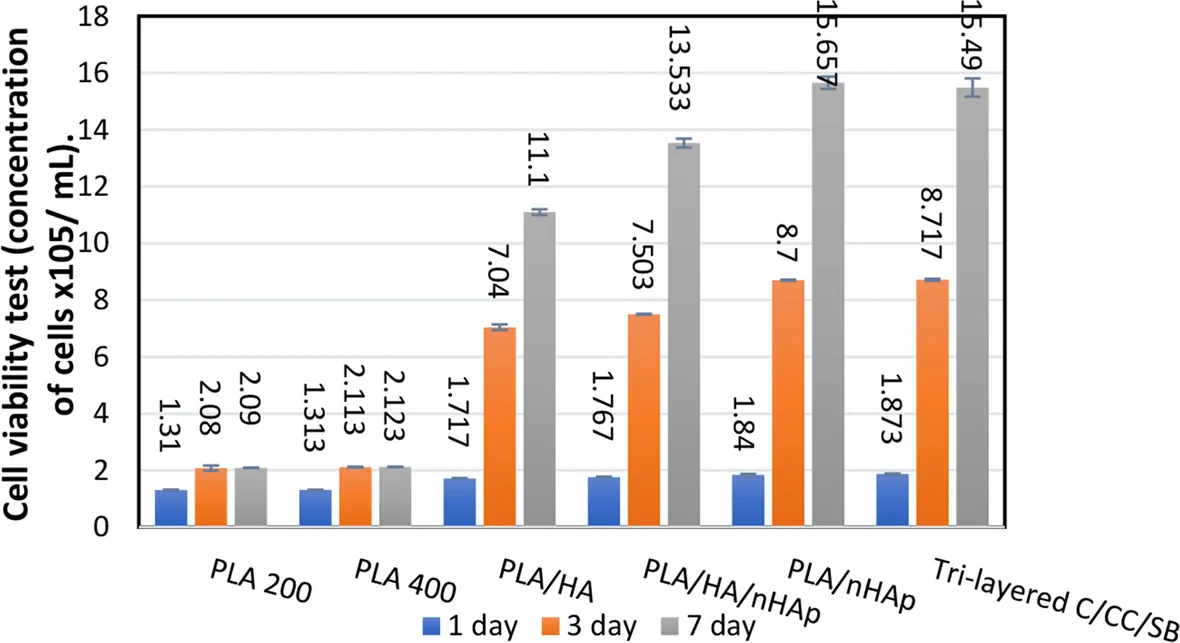
Bar chart showing the mean concentration of cells (cell × 105/mL) and SD (Error bars) values of the groups at different time intervals

Two-way ANOVA revealed statistically significant differences in cell proliferation among the groups (*p* = 0.001) and between aging periods (*p* = 0.001), also the interaction between the two variables was significant at *p* = 0.001 (Fig. 11).

Regarding PLA 200 and PLA 400 groups, the cell concentration at 3 days was higher than at 1 day, while there were no significant differences between 3 and 7 days. PLA/HA cartilage, PLA/HA/nHAp CC, PLA/nHAp SB, and tri-layered C/CC/SB groups showed increased cell concentration from 1 to 7 days, with significant differences between the three intervals.

### Cell adhesion

**Fig. 12:**
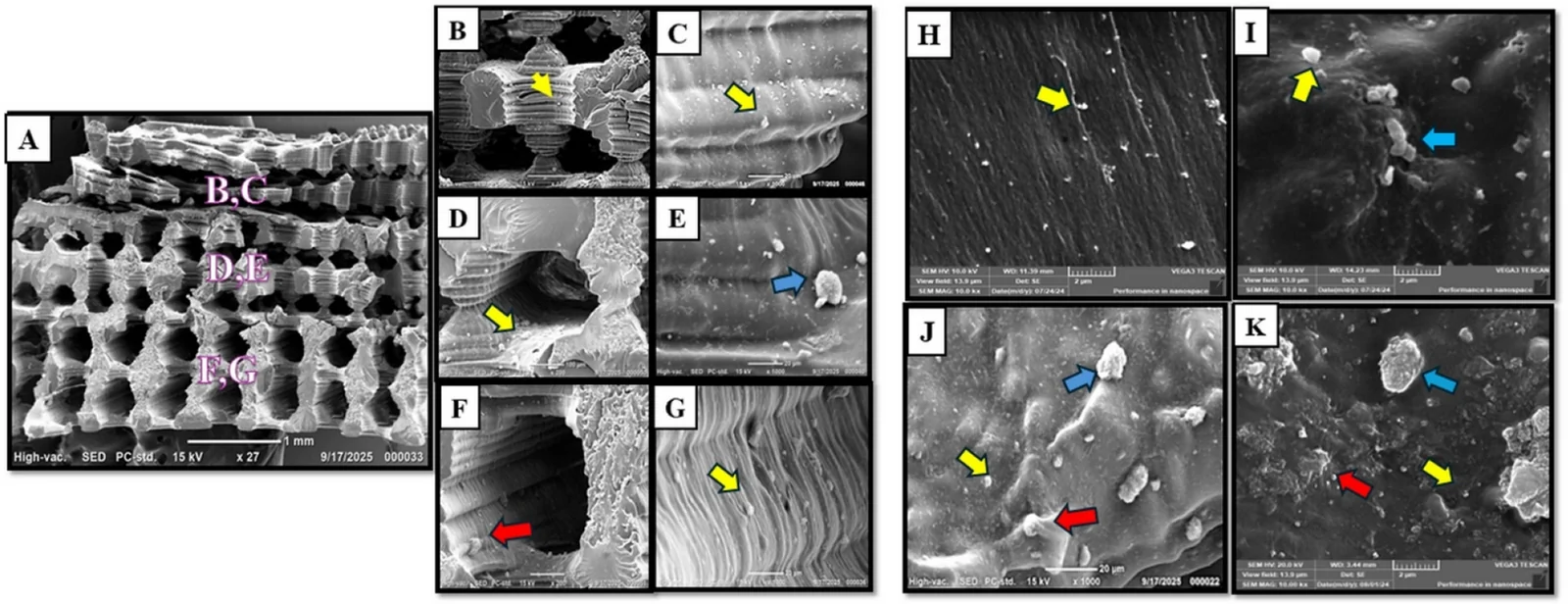
**A** Scanning electron photomicrograph of the tri-layered scaffold cocultured with rBMMSCs for 7 days, revealing the three different layers of the scaffold: **B**, **C** High magnifications for the top cartilage layer, revealing attached cells(yellow arrows) on the scaffold and within the pores, **D**, **E** High magnifications of the intermediate CC layer, revealing surface-associated structures consistent with cell attachment (yellow arrows) and scaffold colonization (blue arrows), **F**, **G** High magnifications of the SB layer, revealing surface-associated structures consistent with cell attachment (yellow arrows) and extending cellular processes in some areas (red arrows). **H** Scanning electron photomicrograph of PLA scaffold, showing surface-associated structures consistent with cell attachment (yellow arrows), **I** PLA/HA cartilage scaffold, showing attached cells (yellow arrows); **J** PLA/HA/nHAp CC scaffold, showing attached cells (yellow arrows), formation of cell colonies (blue arrows), and extending cellular processes in some areas (red arrows); **K** PLA/nHAp SB scaffold, showing attached cells with extending cellular processes (red arrows) and formation of cell colonies (blue arrows)

Scanning electron photomicrographs of the single-layered and tri-layered scaffolds after 7 days of coculturing with rBMMSCs showed morphological features suggestive of cell-scaffold interaction (Fig. 12).

### Hematoxylin & eosin staining

**Fig. 13:**
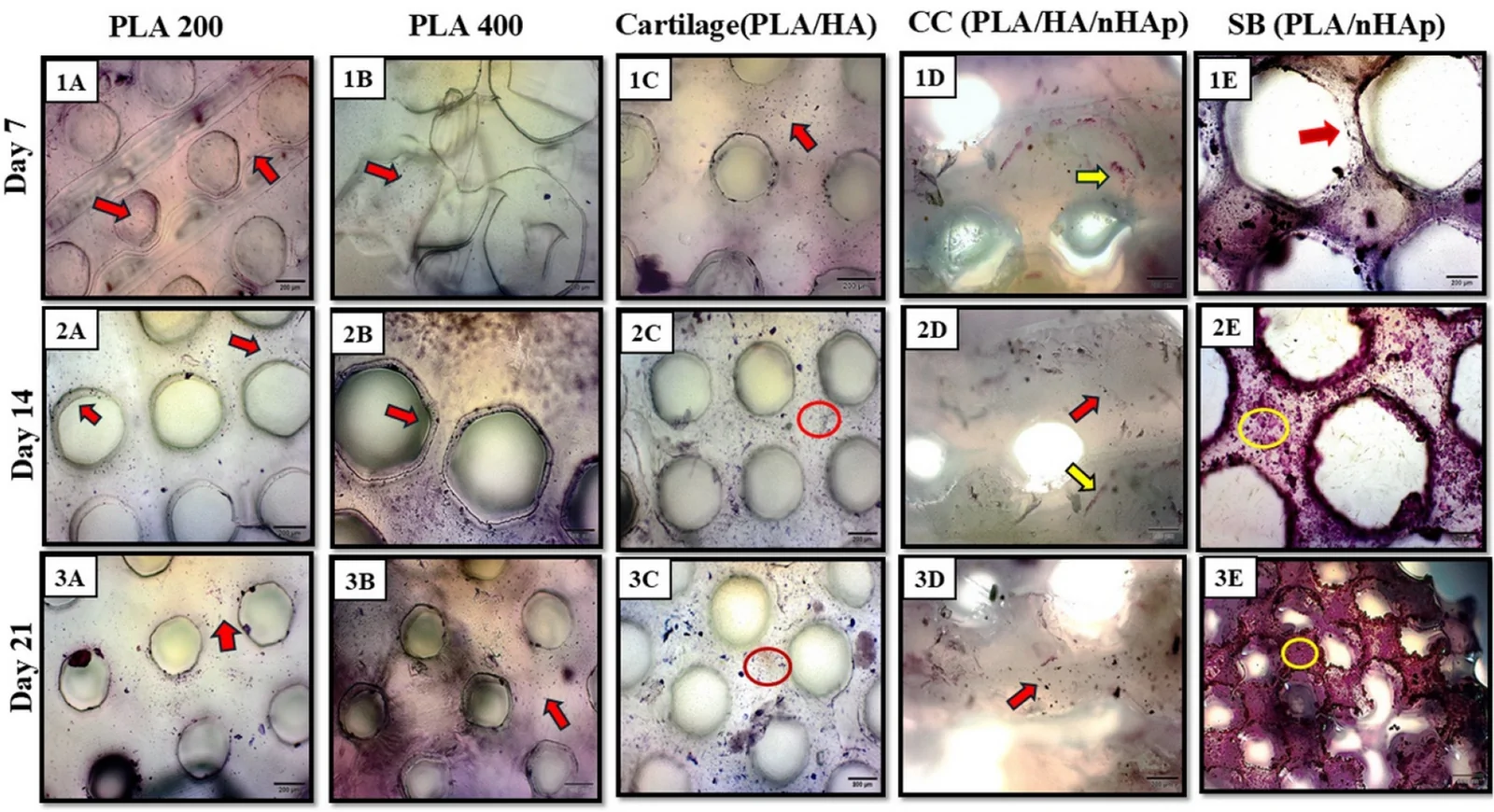
Photomicrographs of H&E-stained sections of single-layered scaffolds after coculturing with rBMMSCs (scale bar of 200 µm), 1 A, 2 A, 3A. PLA 200 group, showing basophilic-stained elongated nuclei infiltrating the pores and scattered over the scaffold structure (red arrows) at 7, 14, and 21 days, respectively, 1B, 2B, 3B. PLA 400 group at 7, 14, and 21 days, respectively, 1 C, 2 C, 3C. PLA/HA cartilage group, showing a more uniform and dense distribution of cells over the scaffold structure and around the pore margins (red circle) at 7, 14, 21 days, respectively, 1D, 2D, 3D. PLA/HA/nHAp CC group, showing attached basophilic-stained nuclei distributed over the scaffold structure (red arrows) with the appearance of eosinophilic ECM -stained areas (yellow arrows), 1E, 2E, 3E. PLA/nHAp SB group, showing uniform distribution of cells over the scaffold structure and pore margins with eosinophilic ECM deposition in multiple areas (yellow circle) at 7, 14, and 21 days, respectively

The hematoxylin &eosin-stained photomicrographs showed progressive cellular infiltration and proliferation over time. They showed the attachment of rBMMSCs on the scaffold surfaces and around the pore walls. The cells displayed elongated, basophilic nuclei, typical for mesenchymal stem cells, with the appearance of eosinophilic extracellular like matrix (ECM)-stained areas in PLA/HA/nHAp CC and PLA/nHAp SB groups (Fig. 13).

### Chondrogenic differentiation results

#### Fast green/safranin O staining

**Fig. 14:**
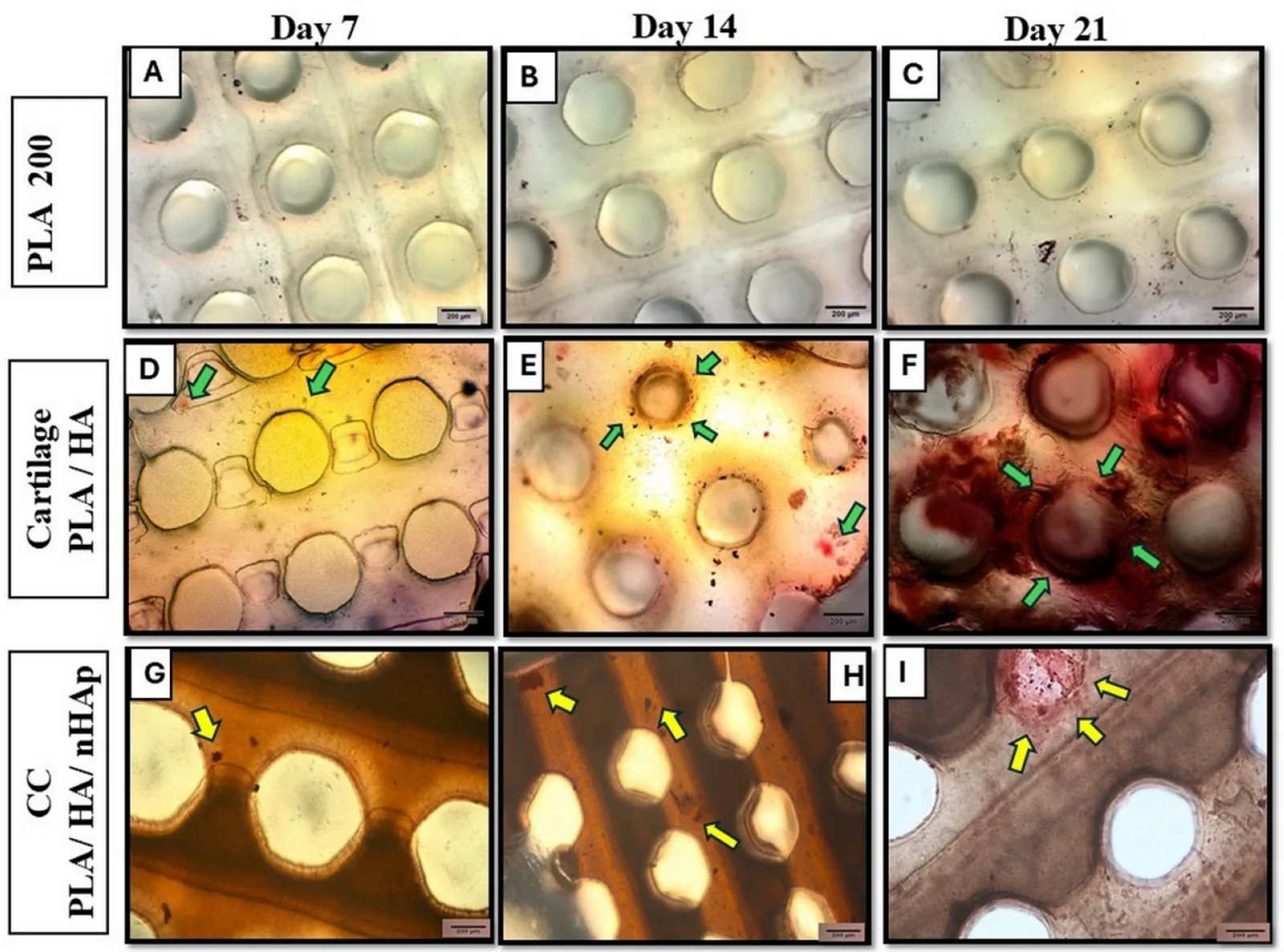
Photomicrographs of fast green/S.O stained sections after coculturing with rBMMSCs: **A**, **B**, **C** PLA 200 scaffold at 7, 14, and 21 days, respectively, showing negative staining, **D** Cartilage scaffold after 7 days, showing positive-stained deposits on the scaffold (green arrows), **E** The cartilage after 14 days, showing positive staining in multiple areas (green arrows). **F** Cartilage group at 21 days, revealing wide positive areas with occlusion of multiple pores (green arrows), **G**, **H**, **I** CC group at 7, 14, and 21 days, respectively, showing positive-stained deposits on the scaffold with occlusion of some pores at 21 days (yellow arrows)

**Fig. 15:**
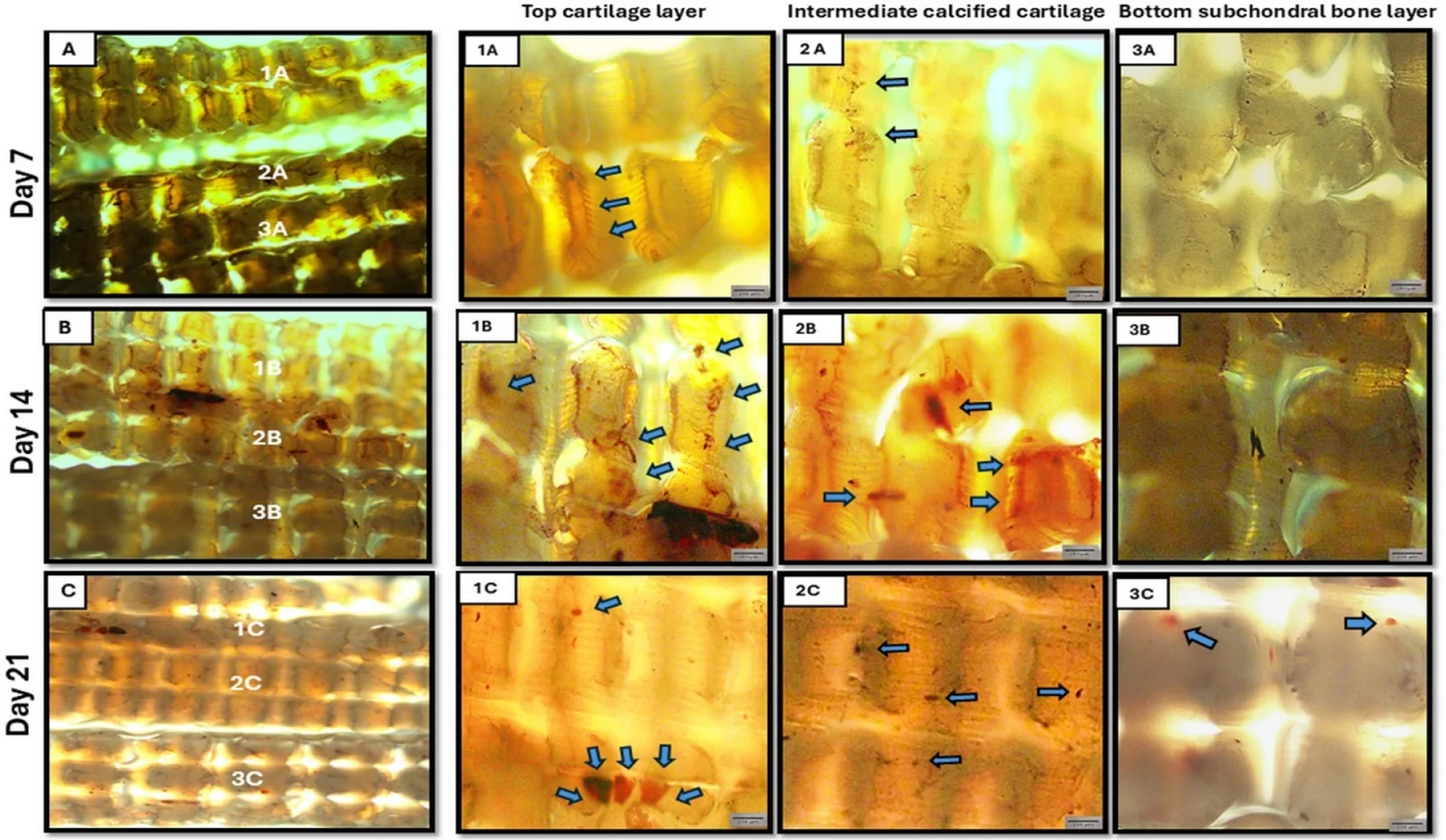
Photomicrographs of fast green/S.O-stained sections of the tri-layered scaffold: **A** Tri-layered structure overview at 7 days, 1A. The top cartilage layer, showing positive staining, 2A. The intermediate CC layer, showing positive staining, 3A. The bottom SB layer, showing negative staining, **B** Tri-layered structure overview at 14 days, 1B. The top layer, showing positive-stained deposits, 2B. The intermediate layer, showing positive staining in multiple areas, 3B. The bottom layer, showing negative staining, **C** Tri-layered structure overview at 21 days, 1C. The top layer, showing positive staining with the formation of large nodules of deposited matrix, 2C. The intermediate layer, showing positive staining, 3C. The bottom layer, showing positive staining at a few points. Blue arrows denote positive fast green/S.O staining

Fast green/safranin O positive staining was seen in the single-layered PLA/HA cartilage and PLA/HA/nHAp CC groups, unlike the PLA 200 control group, which showed negative staining. On extending the culturing duration, staining covered wider areas, and occlusion of some pores with deposited matrix was observed in the cartilage group, as shown in Fig. 14. In the tri-layered C/CC/SB scaffold, the section integrating all three layers showed that the top cartilage layer exhibited positive staining, which gradually decreased along the section to reach a minimum at the bottom SB layer, suggesting the capability of the top and intermediate layers to deposit the glycosaminoglycans cartilaginous matrix as shown in Fig. 15.

#### Aggrecan immunofluorescence results

**Fig. 16:**
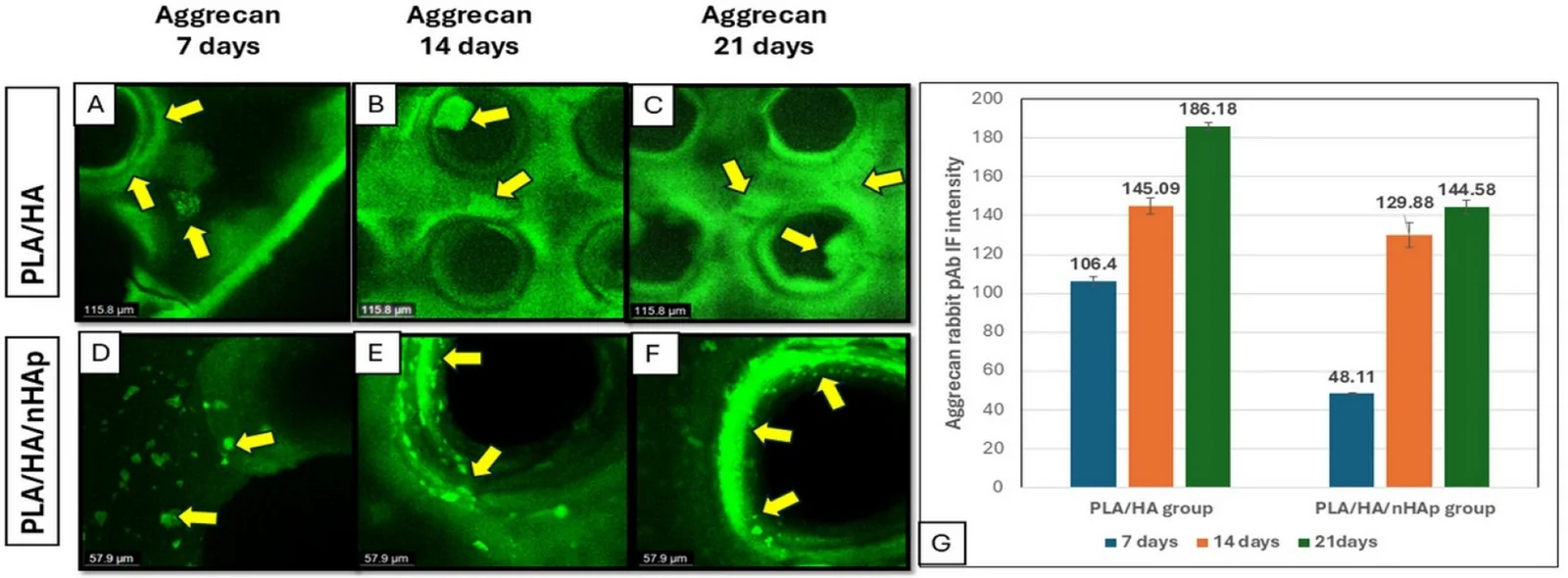
**A**, **B**, **C** Photomicrographs of CLSM images of the PLA/HA cartilage group stained with Aggrecan rabbit pAb antibody at 7, 14, and 21 days, respectively, showing the increased aggrecan-associated fluorescence over time that appeared as IF nodules inside and around the pores (yellow arrows), **D**, **E**, **F** Photomicrographs of CLSM images of PLA/HA/nHAp CC group stained with Aggrecan rabbit pAb antibody at 7, 14, and 21 days, respectively, showing the increased aggrecan-associated fluorescence over time that appeared as IF areas on the scaffold and at the edges of the pores (yellow arrows), **G** Bar chart of the mean IF intensity for aggrecan rabbit pAb and SD (Error bar) of PLA/HA and PLA/HA/nHAp groups at 7, 14, and 21 days. Representative negative control of aggrecan is presented in additional file 3

**Fig. 17:**
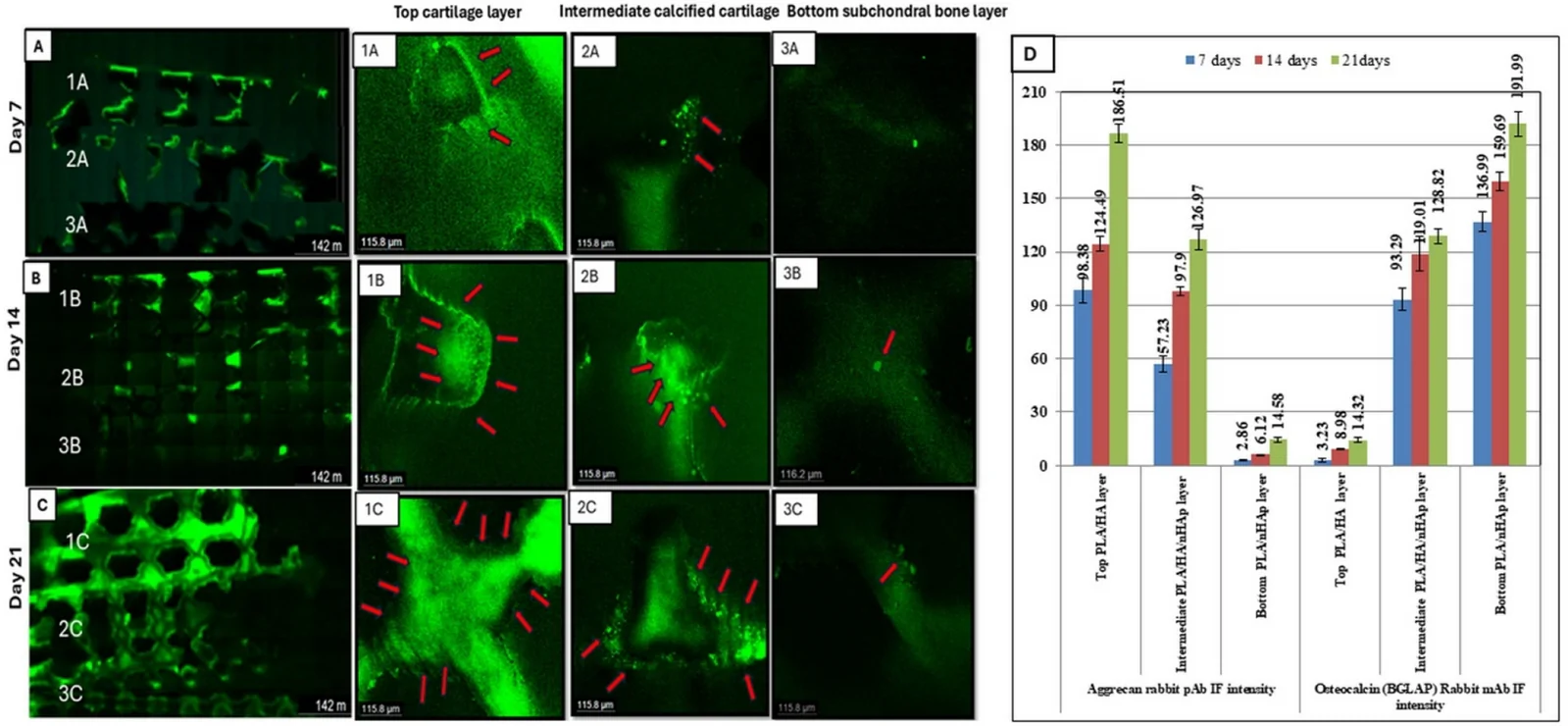
Photomicrographs of CLSM images of the tri-layered scaffold with IF aggrecan: **A** Tri-layered structure overview at 7 days, 1A. The top cartilage layer, showing positive IF areas at the edges of the pores, 2A. The intermediate CC layer, showing positive IF deposits, 3A. The bottom SB layer, showing negative reaction, **B** Tri-layered structure overview at 14 days, 1B. The top layer, showing high positive areas around and at the edges of the pores, 2B. The intermediate layer, showing positive aggrecan at the edges of the pores, 3B. The bottom layer, showing scarce areas of positive reaction, **C** Tri-layered structure overview at 21 days, 1C. The top layer, showing a positive reaction, covering almost all the scaffold, 2C. The intermediate layer, showing positive IF areas, covering multiple areas, 3C. The bottom layer, showing a positive reaction in few areas. Red arrows denote positive IF aggrecan-associated fluorescence. **D** Bar chart showing the mean of IF expression of Aggrecan and Osteocalcin, and SD (Error bar) of the different layers of the Tri-layered C/CC/SB scaffold at 7, 14, and 21 Days

Two-way ANOVA showed significant differences in the immunofluorescence intensity between different groups and different time intervals. PLA/HA cartilage group showed significantly higher aggrecan IF intensity than the PLA/HA/nHAp CC group. Both groups showed an increased pattern from 7 to 21 days, as shown in Fig. 16. Regarding the tri-layered C/CC/SB scaffold, the IF analysis showed significant differences between the scaffold layers at each time interval and within each layer over time (*p* < 0.05). At the 7, 14, and 21 days, the cartilage layer exhibited the highest aggrecan-associated fluorescence, while the SB layer demonstrated the least aggrecan-associated fluorescence, as shown in Fig. 17.

### Osteogenic differentiation results

#### Alizarin red staining

**Fig. 18:**
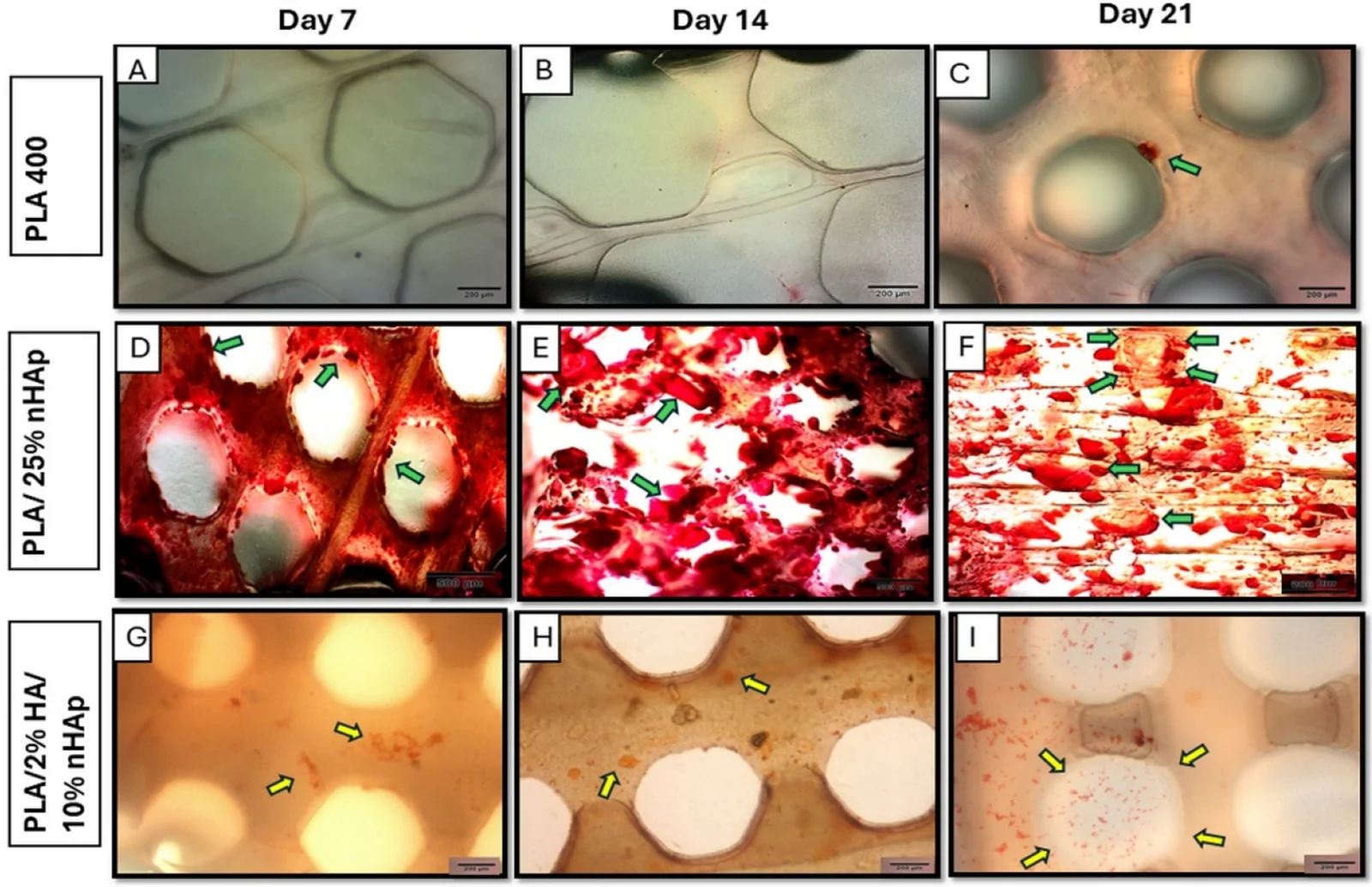
Photomicrographs of ARS-stained sections after coculturing with rBMMSCs: **A**, **B** PLA 400 at 7 and 14 days, showing negative ARS, **C** PLA 400 after 21 days, revealing positive ARS at few areas (green arrow), **D** PLA/nHAp SB after 7 days, showing positive ARS at the edges of the pores (green arrows), E. The PLA/nHAp SB after 14 days, showing positive multiple stained nodules over the scaffold structure (green arrows), **F** The PLA/nHAp SB at 21 days, revealing occlusion of multiple pores (green arrows), **G**, **H**, **I** PLA/HA/nHAp CC at 7, 14, and 21 days, respectively, showing positive-stained deposits on the scaffold with occlusion of some pores at 21 days (yellow arrows). A representative negative control image is presented as additional file 2

**Fig. 19:**
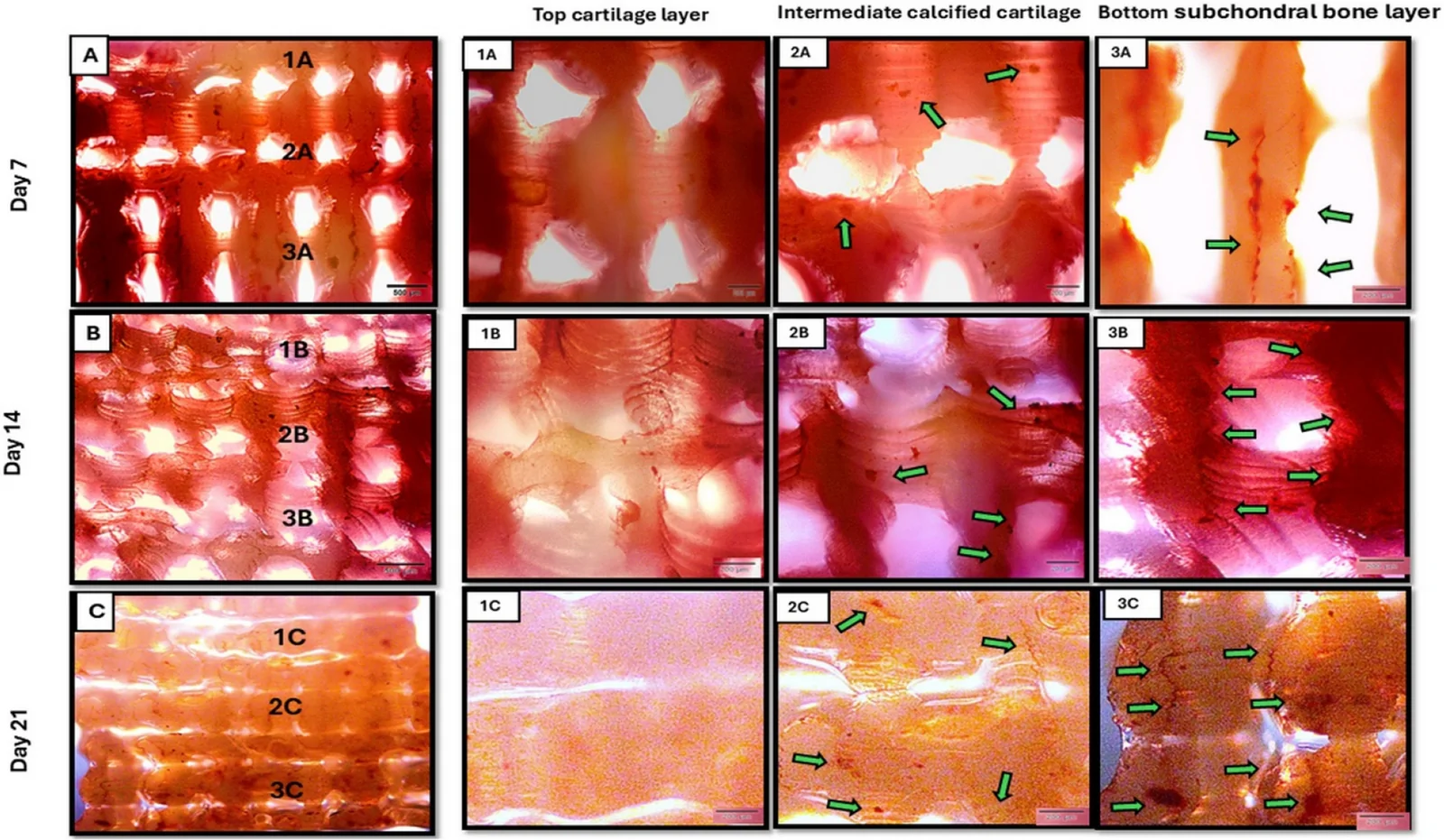
Photomicrographs of ARS-stained sections of the tri-layered scaffold structure: **A** Tri-layered structure overview at 7 days, 1A. The top cartilage layer, showing negative ARS, 2A. The intermediate CC layer, showing positive ARS, appeared as precipitates around the pores, 3A. The bottom SB layer, showing positive ARS, **B** Tri-layered structure overview at 14 days, 1B. The top layer, showing negative ARS, 2B. The intermediate layer, showing positive ARS in multiple areas, 3B. The bottom layer, showing positive ARS over wider areas, **C** Tri-layered structure overview at 21 days, 1C. The top layer, showing negative ARS, 2C. The intermediate layer, showing positive ARS nodules, 3C. The bottom layer, showing positive ARS, with the formation of large nodules. Green arrows denote positive ARS

The PLA/nHAp SB and PLA/HA/nHAp CC groups showed positive ARS, which covered more areas by increasing the culturing time. The PLA 400 group showed negative ARS at 7 and 14 days, while positive staining was revealed at 21 days (Fig. 18). Regarding the tri-C/CC/SB scaffold, the longitudinal section integrating all three layers showed that ARS was positive and intense in the bottom SB layer, gradually decreasing to a minimum in the top cartilage layer, suggesting the capability of the intermediate and bottom layers to deposit the inorganic bone matrix, as shown in Fig. 19. Acellular scaffold control demonstrated baseline staining attributable to scaffold-associated calcium-containing phases (Additional file 2). The distribution and intensity observed in cell-seeded scaffolds exceeded the baseline staining pattern observed in the corresponding acellular controls.

#### Osteocalcin immunofluorescence results

**Fig. 20:**
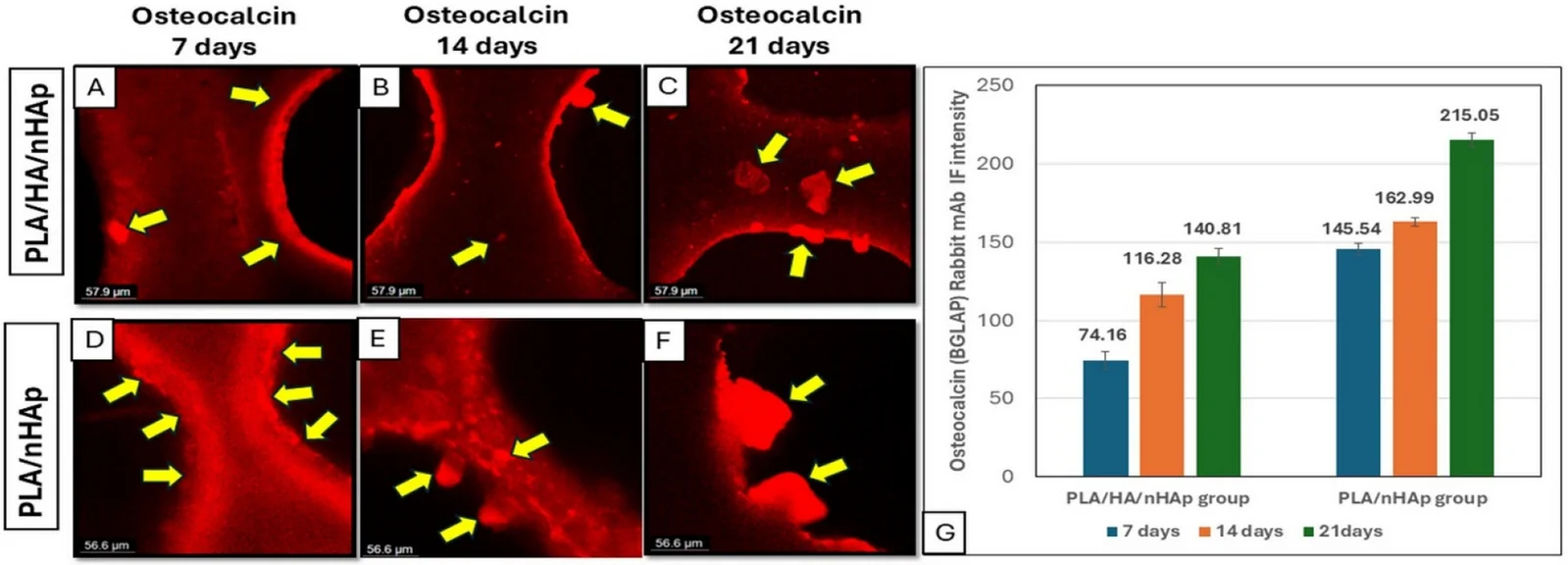
**A**, **B**, **C** Photomicrographs of CLSM images of the PLA/HA/nHAp stained with IF osteocalcin at 7, 14, and 21 days, respectively, showing positive IF areas at the edges of the pores and on the inter-pore areas (yellow arrows), **D**, **E**, **F** Photomicrographs of CLSM images of the PLA/nHAp with osteocalcin antibody at 7, 14, and 21 days, respectively, showing positive IF areas at the edges of the pores and on the inter-pore areas (yellow arrows), **G** Bar chart of the mean IF intensity for osteocalcin and SD (Error bar) of PLA/nHAp and PLA/HA/nHAp groups at 7, 14, and 21 days

**Fig. 21:**
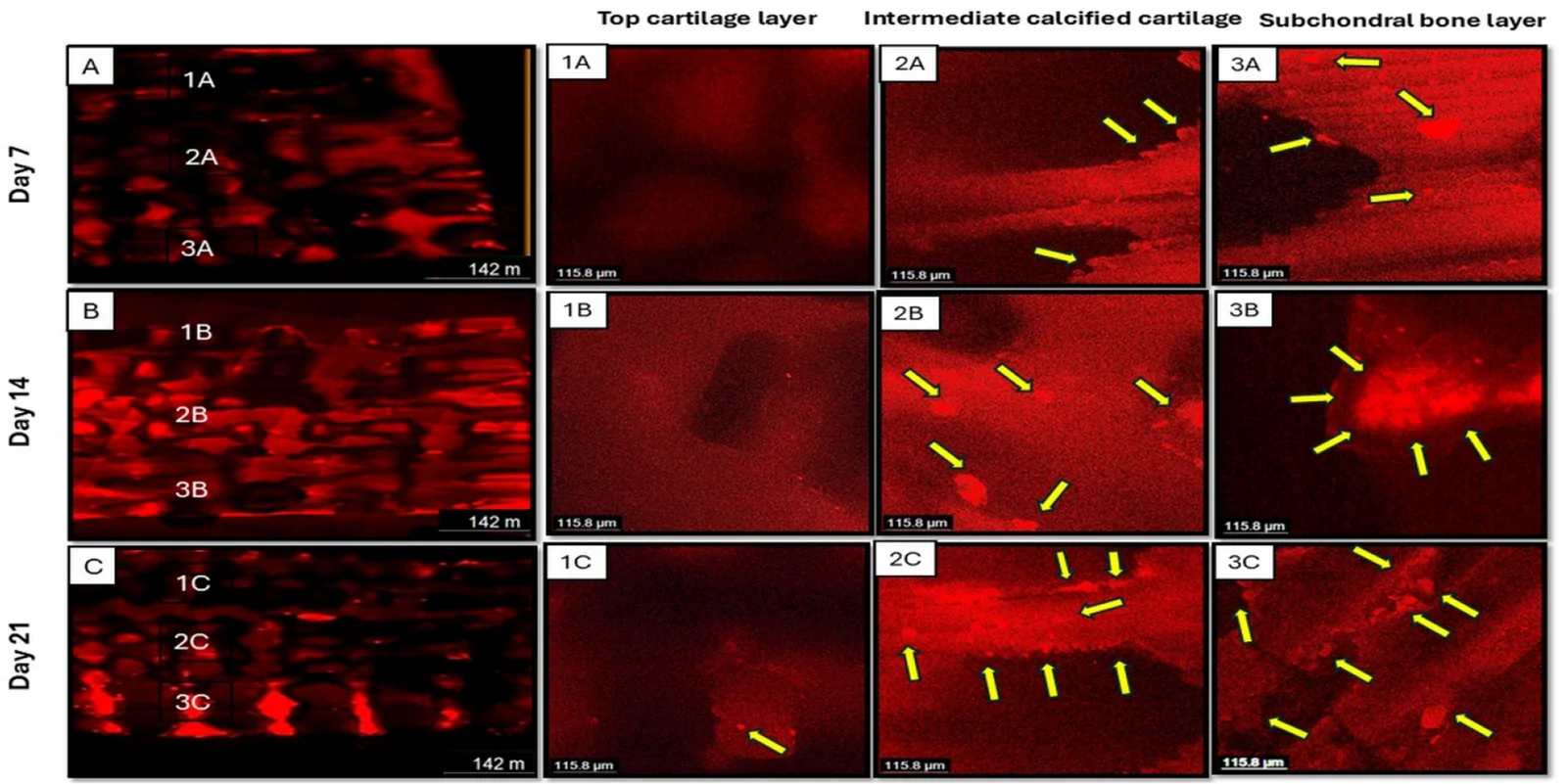
photomicrographs of CLSM images of the tri-layered scaffold with IF Osteocalcin: **A** Tri-layered structure overview at 7 days, 1A. The top cartilage layer, showing a negative reaction, 2A. The intermediate CC layer, showing positive small IF nodules on the scaffold and at the pores, 3A. The SB layer, showing large nodules over the scaffold, **B** Tri-layered structure overview at 14 days, 1B. The top layer, showing negative IF, 2B. The intermediate layer, showing positive IF in multiple areas, 3B. The bottom layer showing positive IF aggregates covering the inter-pore area, **C** Tri-layered structure overview at 21 days, 1C. The top layer, showing a positive reaction in a few areas, 2C. The intermediate layer, showing positive IF nodules scattered over the scaffold, 3C. The bottom layer, showing positive aggregated nodules with partial occlusion of some pores. N.B: Yellow arrows denote positive osteocalcin- associated fluorescence. Representative negative control is presented in additional file 4

The PLA/nHAp SB group showed significantly higher osteocalcin IF intensity than the PLA/HA/nHAp CC group. The two groups demonstrated an increased pattern from 7 to 21 days, as shown in Fig. 20. Regarding the tri-layered C/CC/SB scaffold. The IF analysis showed significant differences between the scaffold layers at each time interval and within each layer over time (*p* < 0.05). At the 7, 14, and 21 days, the top cartilage layer exhibited the least osteocalcin, while the bottom SB layer demonstrated the highest osteocalcin -associated fluorescence, as shown in Figs. 17 D and 21.

## Discussion

Regenerative medicine offers an alternative treatment for osteochondral (OC) defects, including degenerative conditions of the TMJ [17]. In this study, the first null hypothesis was accepted, and the second null hypothesis was rejected.

The tri-layered scaffold was designed with a porosity gradient to facilitate simultaneous chondrogenic and osteogenic regeneration. The optimal pore size hasn’t been identified yet. Some previous studies have stated that a scaffold with a percentage porosity higher than 50% and a pore size larger than 300 µm is recommended to achieve direct osteogenesis with enhanced vascularization. On the other hand, pore size ranging between 90–120 µm is favorable for chondrogenesis [47]. Other studies suggested that larger pores (400–600 μm) resulted in better bone ingrowth, while small pores (150–250 μm) favored hypoxic conditions and induced chondrogenesis in vivo [48, 49]. In the current study, the PLA/HA cartilage layer was designed with small pore size of 200 μm and 50% porosity to enhance chondrogenesis by promoting cell aggregation and creating a hypoxic environment, preventing mineral deposition. While PLA/nHAp SB was designed with large pore size of 400 μm and 70% porosity to enhance osteogenesis by providing space for stem cell penetration and bone growth, as well as creating high oxygen tension that supports blood vessel growth in vivo. The porosity of the calcified cartilage layer was selected to be intermediate between the two layers [47, 49]. The main purpose of designing the calcified cartilage part is to act as an intermediate layer with intermediate properties to prevent abrupt changes that can occur between the two other layers, which can result in stress concentration at the interfaces and delamination during testing. It also creates a physical barrier against vascular invasion into the cartilage, preventing the ossification of the cartilage layer [17, 49].

The comparison between the designed pore size and percentage porosity of various scaffolds and their actual measurements after 3D printing revealed that all scaffolds experienced a slight decrease in porosity compared to their CAD models, except for the PLA/nHAp group. This group showed a greater decrease, possibly due to the high concentration of nHAp, which might cause partial blockage of some pores. Even after such a decrease, the resulting pore size and % porosity is still within the reported range that can demonstrate regenerative osteogenic potential.

TMJ routinely undergoes compression, shear, and tension loading during normal function. In this study, the compression and tensile properties were evaluated to understand the mechanical behavior of fabricated scaffolds under different loads. The compressive, tensile strength, and moduli of PLA 200 were significantly higher than those of PLA 400. This may be explained by the smaller pores in the PLA 200 group, providing more material per volume, which enhances the mechanical strength and stiffness of the scaffold. This highlights the influence of pore size and % porosity on mechanical properties. Incorporating 2% hyaluronic acid into the PLA in the cartilage scaffold resulted in tensile strength and modulus values after 24 h that resemble the normal condylar cartilage’s tensile strength (2- 10 MPa) and tensile modulus (29 ± 13 MPa) [50]. After 30 days, the mechanical strength and modulus of the PLA/HA increased. HA, an anionic glycosaminoglycan polysaccharide, possesses electron donor groups that bind protons during hydrolysis [51, 52]. Accordingly, the hydrolysis of the HA may expose free sites of carboxylate groups, interacting with the free cations in the PBS, forming polysaccharide–cation complexes that strengthen the scaffold during degradation.

The addition of 25% nano-hydroxyapatite in the PLA/nHAp SB scaffold demonstrated higher mechanical properties than the PLA 400. However, it showed lower compressive and tensile strength compared to the PLA/HA/nHAp. This could be attributed to the combined effect of increasing the pore size to 400 μm, the porosity to 70%, and the brittleness of the nHAp. Additionally, the nHAp particles do not form strong bonds with the PLA matrix, disrupting the continuity of the scaffold [28]. These results were consistent with Wang et al., 2021 [28], and Zhang et al., 2021 [53]. On the contrary, the PLA/nHAp showed higher stiffness compared to PLA 400 and PLA/HA/nHAp, which is required in bone scaffolds to withstand deformation and provide support during cartilage formation. This finding was in line with Pitrolino et al., 2022 [54]. After 30 days, the mechanical properties of the PLA/nHAp decreased, owing to the increased dissolution of nHAp and further weakening. Even after such a decrease, it could still meet the mechanical properties of normal trabecular bone. In which the compressive strength and modulus range from 1—10 MPa and 1—900 MPa, respectively.[47, 50]and the tensile strength is 0.5–10 MPa, and the tensile modulus is 0.05 to 0.5 GPa [47].

Based on the mechanical properties results, we could confirm that the fabricated tri-layered scaffold exhibited a stiffness gradient with the top cartilage layer having the lowest modulus, followed by the intermediate CC layer, and the SB layer had the highest stiffness. Stiffness gradient is important for directing cell differentiation, where chondrogenesis is enhanced on the more flexible scaffold, and osteogenesis occurs on the stiffer one [55].

A key challenge for restoring articulating joint surfaces with 3D printed scaffolds is to withstand delamination or mechanical failure during function. The fabricated tri-layered C/CC/SB scaffold showed no evidence of delamination at the interfaces during the interfacial adhesion strength testing, and the main failure mode was cohesive, indicating strong bonding between the layers. This could be attributed to the use of the PLA photopolymer as the main constituent of all layers, which promoted chemical bonding through its UV polymerization during 3D printing. The advanced tilt-release technology of the used 3D printer may have decreased the separation between the layers of the scaffold during the 3D printing process. There was no statistically significant difference in the pattern of failure mode distribution at 24 h and 30 days’ time interval, indicating that aging in PBS for 30 days did not alter the failure pattern of the scaffold interfaces.

The ability of osteochondral scaffolds to integrate with the surrounding tissue and progressively degrade while being replaced by regenerated tissue is key to cartilage and bone repair applications [56]. The phosphate-buffered saline (PBS) was chosen as the storage medium in the evaluation of mechanical properties and in-vitro biodegradation of the scaffolds because it is a balanced salt solution commonly employed as a body fluid-equivalent medium. PBS has similar dielectric properties to biological tissues and a pH of 7.4. Its buffering capacity helps maintain a stable pH if degradation occurs, ensuring consistent and reliable results [57, 58]. Several papers use 28–30 days as a default endpoint in established protocols. It is considered a critical early phase to follow the biodegradation and mechanical stability of the scaffolds [59–62].

The PLA control groups demonstrated negligible weight loss % over the 30 days of the study. This finding was closely similar to Zernitckaia et al., 2025 [63], who reported that the PLA scaffold remained largely intact after one month in PBS. Incorporating 2% hyaluronic acid into the PLA/HA cartilage layer sped up PLA's degradation. This could be attributed to its hydrophilicity due to the presence of amine, hydroxyl, and carboxyl groups, increasing the polarity of the scaffold, facilitating the formation of hydrogen bonds with water, and in turn promoting its hydrolysis [64]. This result was consistent with Farsi et al., 2022 [64].

It was confirmed that the addition of nHAp to the CC (PLA/HA/nHAp) and SB (PLA/nHAp) scaffolds led to their biodegradation when immersed in PBS. The degradation of nHAp occurs through hydrolysis. The nHAp particles gradually dissolve or detach from the scaffold’s matrix when immersed in PBS. This dissolution process significantly contributes to the scaffold’s degradation [65]. In this study, the CC scaffold showed a higher weight loss percentage at 30 days compared to the SB scaffold. This may be attributed to the combined effect of hyaluronic acid with nHAp in the scaffold's degradation.

Based on the biodegradation results, we could confirm that the tri-layered scaffold exhibited a biodegradation gradient. In which the cartilage layer exhibited the highest weight loss after 30 days. It degraded faster than the intermediate CC and the bottom SB layers. These findings are consistent with the faster cartilage regeneration compared to the underlying bone, which requires support from the bone during its regeneration [66]. We could conclude that the degradation had no significant effect on the mechanical properties of the PLA/HA, PLA/HA/nHAp, and tri-layered scaffolds over the 30 days, which is critical for the clinical suitability of the scaffolds, while degrading without any impact on scaffold properties [56].

In the current study, rat bone marrow mesenchymal stem cells (rBMMSCs) were used for osteochondral regeneration due to their multipotency. They can replicate as undifferentiated cells and can differentiate into multiple tissues [2]. Cell proliferation results demonstrated progressive cell proliferation over time, which confirmed the biocompatibility of the scaffolds and emphasized the effect of scaffold composition on cellular activity. The PLA/HA scaffold showed higher cell proliferation compared to the PLA 200 control group. This could be attributed to the hydrophilicity of the hyaluronic acid, directly enhancing initial cell attachment and facilitating cell proliferation and growth [64].

The incorporation of nHAp particles in the SB and CC scaffolds improved the cell proliferation over time. This could be due to the increased hydrophilicity caused by the presence of the hydroxyapatite particles, which have hydroxyl and phosphate groups [53, 67]. Additionally, the size of hydroxyapatite nanoparticles significantly influences hydrophilicity; smaller particles have a larger surface area, allowing better cellular attachment and proliferation [67]. It was observed that the PLA/nHAp scaffold exhibited higher cell proliferation than the PLA/HA/nHAp, which could be attributed to the increased nHAp concentration to 25%, the increasing pore size (400 µm) and percentage porosity (70%), facilitating more stem cell penetration and proliferation.

In this study, the PLA/HA cartilage showed positive fast green/S.O staining compared to PLA 200, which suggests the scaffold's ability to deposit GAG and promote cartilage formation without the need for chondrogenic growth factors. This effect was attributed to the HA, which is a major component of cartilage’s extracellular matrix (ECM) and can link aggrecan molecules to larger proteoglycans, thus promoting chondrogenesis [11, 18]. These results were consistent with You Shie et al., 2017 [68].

The PLA/nHAp SB demonstrated positive ARS compared to the PLA 400, suggesting a greater level of mineralization, which was a sign of late-stage osteogenic differentiation [38]. This suggests the ability of the SB scaffold to promote osteogenesis even without osteogenic growth factors. This could be explained by the dissolution of the nHAp, into calcium and phosphate ions. Calcium is known to support cell adhesion, proliferation, differentiation, and ECM mineralization, while phosphate regulates cell proliferation and stimulates the expression of key proteins involved in the mineralization process [42, 69]. These findings were consistent with Marcela P. Bernardo et al., 2022 [42]. The PLA/HA/nHAp group showed positive results for both ARS and fast green/S.O staining, showing signs of both cartilage and bone, due to the inclusion of both HA and nHAp, supporting its function as an intermediate layer, The results of the histochemical staining of the tri-layered C/CC/SB scaffold suggesting the capability of each layer of the tri-layered scaffold to direct the formation of different tissues of the osteochondral complex.

Immunofluorescence (IF) analysis of the fabricated scaffolds for aggrecan and osteocalcin proteins confirmed the findings of the histochemical staining. It was demonstrated that rBMMSCs could show a tendency toward differentiation into specific cell types in different scaffolds. The PLA/HA cartilage scaffold predominantly showed aggrecan- associated fluorescence, suggesting its ability to deposit aggrecan protein for cartilage -like tissue formation and differentiation of rBMMSCs into chondrocytes without the need for chondrogenic growth factors. This effect was attributed to the addition of hyaluronic acid to the PLA, which is a major component of cartilage’s extracellular matrix (ECM) and can link aggrecan molecules to larger proteoglycans. Hyaluronic acid plays a key role in regulating cell functions, including promoting chondrogenic phenotypes, as well as the production and retention of matrix components [11, 70, 71].

Whereas the PLA/nHAp SB scaffold predominantly showed osteocalcin- associated fluorescence, suggesting the formation of osteoid tissue and the differentiation of rBMMSCs into osteoblasts. This indicates that the 3D printed subchondral bone scaffold could potentially contribute to osteogenesis even without osteogenic growth factors. This could be based on the presence of nHAp that was included in the SB layer, where the dissolution of nHAp into calcium and phosphate ions plays an essential role in bone formation. It has been reported that calcium supports cell adhesion, proliferation, differentiation, and ECM mineralization, while phosphate stimulates the expression of key proteins involved in the mineralization process [7]. These findings were consistent with Marcela P. Bernardo et al., [42].

The calcified cartilage scaffold showed aggrecan- and osteocalcin-associated fluorescence, which is consistent with its role as a calcified cartilage interface. Additionally, expression levels of all scaffolds increased gradually from 7 to 21 days, reflecting ongoing scaffold bioactivity. Based on the previous findings, we can emphasize the significance of incorporating the calcified cartilage layer as an intermediate layer between the cartilage and subchondral bone layer. From a biological perspective, the CC functions as a transitional interface between cartilage and subchondral bone (SB), as the immunofluorescence and histochemical staining provided evidence of the deposition of both cartilaginous and mineralized matrices in the CC scaffold. Mechanically, the calcified cartilage (CC) exhibited intermediate rigidity values, which aided in transferring load from the cartilage to the bone without delamination, as demonstrated in the interfacial adhesion strength assessment. The CC scaffold was specifically engineered to simulate these structural and functional attributes. It was designed with an intermediate pore size, a specific percentage of porosity, and a graded mineral content of 10% nano-hydroxyapatite and 2% hyaluronic acid, trying to replicate the partial mineralization characteristic of the calcified cartilage layer with formation of some cartilaginous matrix. These results suggested the potential of each layer of the tri-layered scaffold toward the formation of different tissues of the osteochondral complex.

## Conclusions

The proposed fabrication technique successfully produced biomimetic single-layered scaffolds exhibiting tensile strength and elastic modulus values comparable to those reported for native tissues. The fabricated scaffolds supported biological responses consistent with chondrogenic, calcified cartilage-like, and osteogenic differentiation in the absence of lineage-specific growth factor supplementation. Furthermore, the tri-layered scaffold demonstrated strong interfacial adhesion between adjacent layers and resisted delamination under mechanical loading conditions relevant to osteochondral tissues. The scaffold also exhibited physicomechanical gradient properties that mimic the functional transitions present within the native osteochondral complex. Collectively, these findings demonstrate the potential of the fabricated scaffolds as promising candidates for osteochondral tissue engineering and warrant further molecular and in vivo investigations to confirm their regenerative performance and translational potential.

## Limitations

The histological analyses of the tri-layered scaffold were performed using liquid-nitrogen-assisted longitudinal sectioning rather than conventional microtome-generated thin sections because the scaffold material did not tolerate standard paraffin processing without substantial structural loss. While this approach preserved scaffold integrity, it provides lower histological resolution than conventional paraffin or resin sections. Consequently, the staining results should be interpreted primarily as evidence of staining distribution and scaffold-associated biological events rather than definitive histopathological characterization of newly formed tissues.

Immunofluorescence analysis was used to evaluate the localization and relative fluorescence intensity of aggrecan- and osteocalcin-associated extracellular matrix components within the scaffolds. As nuclear counterstaining was not incorporated, the analysis was not intended to assess marker expression on a per-cell basis or normalize fluorescence to cell number. Additional molecular characterization such as RT-qPCR, Western blotting, or expanded marker analyses would provide stronger confirmation. Furthermore, in vivo investigations are required to determine the functional regenerative performance of the scaffold under physiological conditions.

## Supplementary Information

Additional file 1: Representative JPG image showing flow cytometry of rBMMSCs.

Additional file 2: Representative JPG image showing Alizarin Red staining negative control.

Additional file 3: Representative JPG image showing negative control Aggrecan staining.

Additional file 4: Representative JPG image showing negative control Osteocalcin staining.

## Data Availability

The datasets generated and analyzed during the current study are included in this article. All materials used in this study are mentioned in the Materials and Methods section. Additional raw data are available from the corresponding author upon reasonable request via shaimaahamdy@dent.asu.edu.eg.
